# MIP-3α-antigen fusion DNA vaccine enhances sex differences in tuberculosis model and alters dendritic cell activity early post vaccination

**DOI:** 10.1038/s41598-025-06532-6

**Published:** 2025-07-01

**Authors:** James T. Gordy, Rowan E. Bates, Elizabeth Glass, Jacob Meza, Yangchen Li, Courtney Schill, Alannah D. Taylor, Tianyin Wang, Fengyixin Chen, Khaleel Plunkett, Styliani Karanika, Petros C. Karakousis, Richard B. Markham

**Affiliations:** 1 Department of Molecular Microbiology and Immunology, Johns Hopkins School of Public Health, Baltimore, MD USA; 2 Department of Medicine, Division of Infectious Diseases, Center for Tuberculosis Research, Johns Hopkins University School of Medicine, Baltimore, MD USA

**Keywords:** *Mycobacterium tuberculosis* (*Mtb*), Immunology sex differences, Antigen presenting cell (APC), Dendritic cell (DC), DNA vaccine, MIP-3α (CCL20), Vaccines, DNA vaccines, Antigen-presenting cells, Tuberculosis, Immunology, Dendritic cells

## Abstract

Tuberculosis disease (TB) caused by *Mycobacterium tuberculosis* (*Mtb*) bacteria remains a major cause of global morbidity and mortality. Efforts to control TB are hampered by the lengthy and cumbersome treatment required to eradicate the *Mtb* infection. Bacterial persistence during exposure to bactericidal antibiotics is at least partially mediated by the bacterial stringent response enzyme, Rel_Mtb_. A therapeutic DNA vaccine targeting Rel_Mtb_ has been shown to increase the efficacy of antitubercular drugs, and fusing macrophage-inflammatory protein 3α (MIP-3α), which interacts with CCR6 on immature dendritic cells (iDCs), to Rel_Mtb_ further increases the vaccine’s therapeutic efficacy. A secondary analysis of these prior studies elucidated prominent sex-based differences in vaccine therapeutic efficacy, with female mice showing improved microbial outcomes compared to males as a result of the Rel and MIP-3α-Rel vaccine constructs, with a more pronounced sex-associated difference in the MIP-3α-Rel group. In the current study, we addressed the hypothesis that these sex-related differences are at least in part due to differential DC activation/function soon after vaccination. An EαGFP reporter vaccine model was used to track vaccine antigen presentation in the draining node with flow cytometry panels by an antibody Y-Ae which binds the Eα peptide tag in complex with I-A^b^ MHC-II molecules. Additionally, a qRT-PCR panel assessing DC-related genes compared sexes receiving the MIP-3α-Rel vaccine. MIP-3α-EαGFP groups had more DCs presenting vaccine antigen infiltrating from the periphery, with more abundant Langerhans cells in males and greater CD8+ CD103+ cross-presenting dermal DCs in females. This model also shows there is greater DC activation, as measured by CD80 and MHC II MFI, by MIP-3α compared to EαGFP alone, especially in female mice. The genetic panel showed females more enriched for chemokines and genes related to cell movement and cross-presentation. Our findings are consistent with the sex- and MIP-3α-related differences seen in the therapeutic model and supports the hypothesis that in both sexes MIP-3α enhances vaccine uptake and cell activation by peripheral iDCs. Additionally, female mice showed greater levels of antigen presentation, especially in DCs able to cross-present antigen, likely explaining why they had the best outcomes. Further studies are required to understand underlying mechanisms and to link APC results directly to T-cell responses.

**Supplementary Information:**

The online version contains supplementary material available at 10.1038/s41598-025-06532-6.

## Introduction

*Mycobacterium tuberculosis* (*Mtb*) is the leading causative agent of tuberculosis disease (TB). Curative treatment for drug-susceptible TB requires a 6-month-long regimen of multiple antibiotics^1^ since a proportion of the bacteria can downshift their growth and metabolism, becoming tolerant to antibiotics^2–5^. These so-called “persisters” show induction of the stringent response, which is regulated by the (p)ppGpp synthase/hydrolase, Rel_Mtb_^6–8^.

Our laboratory developed a therapeutic DNA vaccine that targets Rel_Mtb_ (termed Rel), which synergizes with the first-line antitubercular drug, isoniazid (INH), leading to significantly enhanced lung bacterial clearance^9^. We optimized the vaccine by fusing *rel*_*Mtb*_ to the gene encoding the chemokine macrophage-inflammatory protein 3α (MIP-3α/CCL20; termed MIP-3α-Rel), which interacts with the CCR6 receptor on immature dendritic cells (iDCs) and T-cells^10^. This chemokine fusion has been shown in multiple studies to enhance the vaccine’s efficacy by specifically targeting vaccine antigen to iDCs^11–17^.

Sex-based differences in outcomes for infectious disease, autoimmunity, cancer, and vaccine efficacy have been widely observed^18–20^. Antigen-presenting cells (APCs), which are essential for adaptive immune response initiation and especially critical in our vaccine model, have known differences in phenotype between females and males. In particular, APCs in females exhibit more efficient phagocytosis^21^ and antigen presentation^22^ under certain conditions. Dendritic cells, which are professional APCs, also demonstrate sex-based differences in immunity. In asthma models, female mice had elevated levels of DCs in the lungs and increased trafficking of DCs to the lymph nodes^22–24^. Additionally, estrogens promote DC differentiation, competence, development, and functional responses^25^.

One challenge in studying the role of DCs in vaccination studies is to show vaccine-specific activity at the draining node without in vitro manipulations. Other groups have created a system termed Eα, where amino acids 52–68 of gene *I-E*^*d*^*α* are utilized as an MHC-II antigen processing reporter, because the processed peptide bound to the I-A^b^ MHC-II molecule inherent in C57Bl/6 mouse APCs interacts with commercially available monoclonal antibody Y-Ae^26,27^. For purposes of this study, we created a reporter vaccine including Eα fused to enhanced green fluorescent protein, both with (MIP-3α-EαGFP) and without (EαGFP) MIP-3α fused upstream.

Since MIP-3α targets antigen to iDCs^11–17^, the central hypothesis of this study is that the sex biases in the immune response at the earliest stages of antigen engagement and DC activity are correlated to the observed differences in vaccine efficacy between males and females. Specifically, we show in this study that MIP-3α-Rel vaccination enhanced the female sex bias seen in Rel vaccination. Further, utilizing the MIP-3α-EαGFP vaccine system, we provide evidence that MIP-3α enhances DC activation with a female bias, and that MIP-3α leads to greater antigen presentation from DCs migrating from the periphery, with female mice showing the highest rates of presentation from CD8+  CD103+ cross-presenting dermal cDC1s that are strongly activated.

## Methods

### Vaccines

Vaccine constructs utilizing the mammalian expression system pSecTag2b were prepared as naked plasmid in 1xPBS, as described in previous publications^10^. The vaccine construct comprised of Eα peptide tag fused to eGFP sequnce (termed EαGFP) was constructed by Genscript (Piscataway, NJ) in a pUC plasmid with parallel design to the current, verified Rel vaccine, with HindIII and KasI sites on the 5’ end and BamHI on the 3’ end. The construct was cloned into our pSecTag2b vaccine plasmid by HindIII and BamHI sites to form the EαGFP vaccine. MIP-3α was cloned into the construct using HindIII and KasI to form the MIP-3α-EαGFP vaccine. Cloning was performed using standard laboratory protocols^10^, and clones were verified by restriction digest gel electrophoresis (Supplementary Fig. 1 A-B) and by insert sequencing (JHMI Synthesis and Sequencing Facility [SSF]). Sequences are listed in Supplemental Data File 1. Blot and gel image without cropping/splicing are provided in Supplementary Fig. 2 for reference.

EαGFP and MIP-3α-EαGFP DNA plasmids were maintained in DH5-α (Thermo Fisher Scientific, Waltham, MA) *E. coli*, grown in large cultures, and extracted utilizing Qiagen (Germantown, MD) endotoxin-free kits. Plasmids were verified for purity, supercoiling, and correctness by insert sequencing (JHMI SSF), Nanodrop (Thermo Fisher Scientific, Waltham, MA) spectrophotometry, and agarose gel electrophoresis. Rel and MIP-3α-Rel vaccines have been used and verified previously^10^.

The EαGFP and MIP-3α-EαGFP vaccines were functionally validated by transfecting HEK293T cells using Lipofectamine 3000 (Invitrogen, Waltham, MA) according to the manufacturer’s protocol. Cell lysates were analyzed by denaturing western blots with anti-C-myc primary (Biolegend, San Diego, CA), alkaline phosphotase-conjugated goat anti-mouse secondary antibody (Jackson Immunoresearch), and NBT-BCIP visualization reagent (Sigma Aldrich, St. Louis, MO), according to previously published protocols^28^ (Supplementary Fig. 1C). Cell media supernatant was concentrated 2× using Microsep Advanced 3 K centrifugal filters (Pall Life Sciences, Port Washington, NY), analyzed by semi-denaturing blotting, and visualized for eGFP signal (excitation peak at 488 nm, emission peak at 513 nm) by a FluorChem Q camera (Alpha Innotech, San Leandro, CA) under Blue (475/42 nm) excitation and a green filter for detection (537/35 nm).

### Animal studies and ARRIVE guidelines

**Fig. 1:**
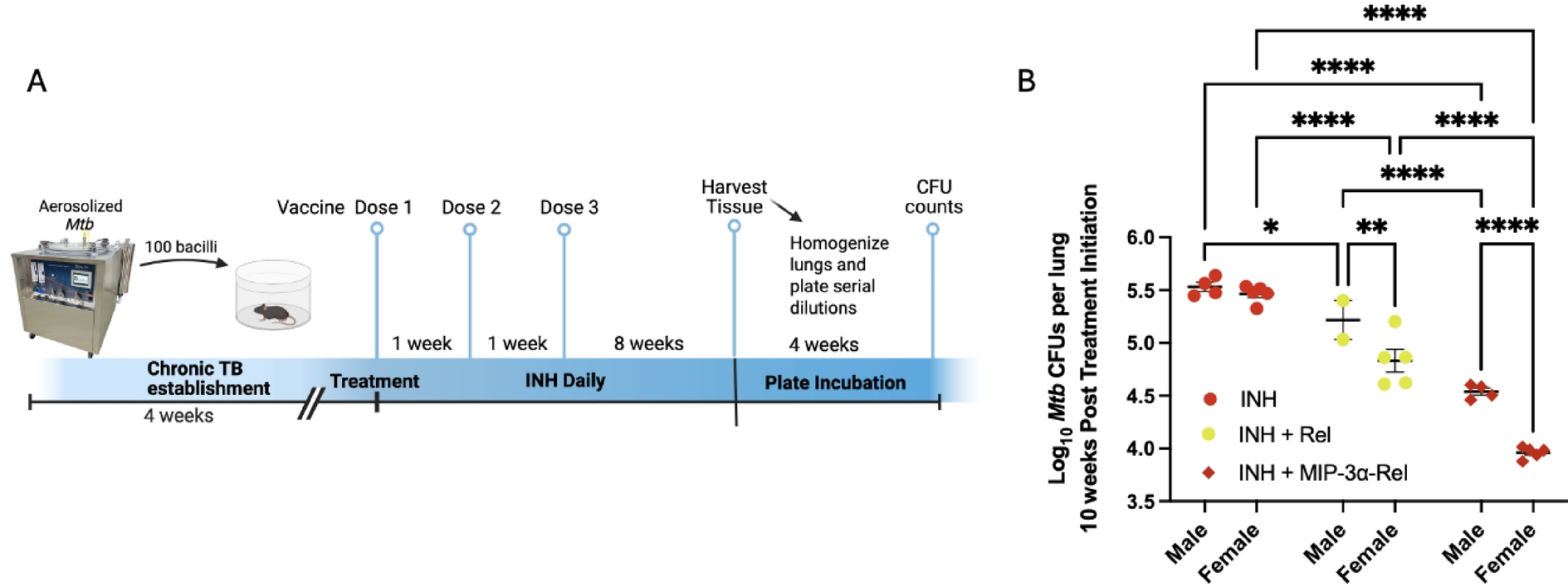
Sex differences of vaccine against *Mycobacterium tuberculosis* (*Mtb*) stringent response protein, Rel_Mtb_. (**A**) Summary of experimental plan as performed by Karanika, et al.^10^ In brief, C57Bl/6 mice were aerosol infected with 100 bacilli of *Mtb* strain H37Rv. 28 days later, mice received daily isoniazid (INH) doses at 10 mg/kg by oral gavage. Mice were vaccinated by intramuscular electroporation three times at one week intervals with Rel or MIP-3α-Rel. At week 10 post treatment initiation, lungs were harvested, homogenized, plated at serial ten-fold dilutions on selective agar, and, after growth, colony forming units (CFU) were calculated. Illustration made using Biorender.com (**B**) Differences in lung bacterial load 10 weeks post treatment initiation of *Mtb*-infected mice receiving INH alone, INH+ Rel vaccine, and INH + MIP-3α-Rel vaccine from previously published dataset^10^, newly stratified by sex of mice. *p < 0.05, **p < 0.01, ****p < 0.0001.

The methodology and experimental design for the study depicted in Fig. 1A were performed previously and have therefore been detailed in a previous publication^10^. In brief, C57Bl/6 mice were aerosol infected with 100 bacilli of *Mtb* strain H37Rv. 28 days later, mice received daily isoniazid (INH) doses at 10 mg/kg by oral gavage. Mice were vaccinated by intramuscular electroporation three times at one week intervals with Rel or MIP-3α-Rel. At week 10 post treatment initiation, lungs were harvested, homogenized, plated at serial ten-fold dilutions on selective agar, and, after growth, colony forming units (CFU) were calculated. Figure 1B is a secondary analysis of the previously published dataset, stratifying select groups by sex. Beyond Fig. 1, the overall study was performed in a 2X2 factorial design testing vaccine composition (with or without MIP-3α) and sex as independent variables. Two independent experiments were performed for a total sample size of 7–8 mice per sex per group. Samples were only excluded in cases of too few cells or experimental error (see Statistics section). Mice were kept in original cages to limit stress, and cages were randomly assigned to groups. Experimental procedures were not performed in any consistent order to limit confounding. Due to a small research team, blinding of samples was not possible.

Outcome measures, statistical methods, experimental methods, and results are described elsewhere in the manuscript. New mouse studies utilized 6–8-week-old C57Bl/6 mice purchased from Charles River Laboratories (Wilmington, MA), purchased as 5–6 weeks old and allowed to acclimate for 1–2 weeks. Due to the short duration of the experiments, weight was not tracked. Male and female mice were anesthetized by vaporized isoflurane and then immunized with saline or 20 μg total of Rel, MIP-3α-Rel, EαGFP, or MIP-3α-EαGFP transfection-grade DNA plasmids injected as 10 μg in 50-μl doses into both mouse hind leg gastrocnemius muscles immediately followed with local electroporation using an ECM830 square wave electroporation system (BTX Harvard Apparatus, Holliston, MA). Each of the two-needle array electrodes delivers 15 pulses of 72 V (a 20-ms pulse duration at 200-ms intervals)^10^. At 48 or 72 h post-vaccination, mice were humanely euthanized by a single dose of 2,2,2-Tribromoethanol (Alfa Aesar/Thermo Scientific) administered intraperitoneally at approximately 250 mg/kg, using 20 g (female) and 25 g (male) as estimated weight, followed by cervical dislocation confirmation after full sedation. All mouse work was conducted using protocols approved by Johns Hopkins University IACUC and in accordance with relevant guidelines and regulations.

### Flow cytometry

Draining popliteal lymph nodes were harvested, and tissue was processed into single-cell suspensions according to laboratory protocols^10,13,28^. Lymph node cells were harvested, processed, and stained on the same day to preserve cell viability. Cells were stained for markers of vaccine antigen protein and presentation (internal eGFP, Y-Ae), DC lineage (CD11c, CD11b, CD8α, B220, CD103, langerin, F4/80), DC trafficking and activation (CCR6, CCR7, CD80, CD86, MHC-II), exclusion of other immune cells (CD3, CD19), and Live/Dead. Antibodies were purchased from either Biolegend (San Diego, CA) or Invitrogen and details of clones, fluorophores, and dilution factors are in Supplemental Table 1. Stained cells were assayed using a Fortessa cytometer (Becton Dickinson, Franklin Lakes, NJ) in the SKCCC Flow Cytometry Technology Development Center (FCTDC). FlowJo (FlowJo, LLC Ashland, OR) software was used for analysis. Gates were determined using Full-Minus-One type staining controls. Since Y-Ae is known to have background signal in mature DCs without Eα^26,27^, Y-Ae gates were further adjusted based on control animals immunized with saline (vehicle), one per sex, to limit background. Gating structure of screening gates are outlined in Supplementary Fig. 3 and gates for specific APC populations are defined in Supplemental Fig. 4A–C.

### qRT-PCR

Mice immunized with Rel or MIP-3α-Rel vaccines had draining popliteal lymph nodes harvested at either day 2 or 3 post-immunization as noted in the figure legends, and the lymph nodes were flash-frozen in RNALater (Invitrogen, Waltham, MA) using a dry/ice + ethanol bath and stored at − 80 °C. Lymph nodes were later thawed and homogenized, and RNA was immediately isolated using TRIzol reagent (Invitrogen, Waltham, MA) according to the manufacturer’s protocols. Day 3 nodal RNA was reverse-transcribed into cDNA by Qiagen’s RT2 First Strand Kit and then assayed using the RT2 Profiler™ PCR Array: Mouse Dendritic & Antigen Presenting Cell, which requires RT2 SYBR Green ROX qPCR Mastermix. The qRT-PCR array was run on the StepOnePlus system (Applied Biosystems, Waltham, MA) in the Department of Molecular Microbiology and Immunology (MMI) Common Equipment Core. Day 2 nodal RNA was reverse-transcribed using SuperScript III First-Strand Synthesis System (Invitrogen, Waltham, MA) and assayed using TaqMan (Thermo Fisher Scientific, Waltham, MA) probes for Rac1 (Mm01201653 mh) and Clec4b2 (Mm02599731 m1) and TaqMan Fast Advanced Master Mix (Applied Biosystems, Waltham, MA) on MicroAmp Fast Optical 96-Well Reaction Plates (0.1 mL) (Applied Biosystems, Waltham, MA). The qRT-PCR assays were run in triplicate using the manufacturer protocol on the Quant Studio 6 Pro Real-Time PCR System (Applied Biosystems, Waltham, MA) in the MMI Common Equipment Core.

### Statistics

Datapoints with < 2 cells were excluded from mean fluorescence intensity (MFI) analysis. One mouse excluded from flow cytometry due to sample processing error. For MFI analyses, to adjust for changes in fluorescent baselines across experiments, baseline measurements of MFI for each parameter (gated on immune cells, single cells, and alive cells) were calculated and averaged per group. Across-experiment shifts were calculated and multiplied to the values of the second experiment to normalize the dataset. For the qRT-PCR Array, 5 mice per group were utilized. Equal total RNA amounts from each mouse per sex/group were pooled to ensure adequate cDNA yield for successful array plate prep and qRT-PCR analysis. For the individual gene qRT-PCR, 4 mice were analyzed per group in triplicate. qRT-PCR Ct values were calculated as the average of the technical triplicates and then normalized to a standard pool of housekeeping gene controls to create the ΔCt statistic. Data were either analyzed as ΔCt or as fold expression difference, calculated as 2^−ΔΔCt^ where ΔΔCt = Female ΔCt – Male ΔCt. All results comparing both group and sex were analyzed by two-way ANOVA with comparisons across rows and columns utilizing Fisher’s LSD Test. Data were stored in Microsoft Excel 2024 (Microsoft Corporation) (Supplemental Data File 2) and analyzed by Prism 10 (GraphPad Boston, MA). α = 0.05.

## Results

### Secondary analysis of therapeutic vaccine study by sex of mice

Our previous *Mtb* mouse challenge study established chronic *Mtb* infection and then three vaccinations were administered at one week intervals while giving daily isoniazid antibiotic (INH) by oral gavage. Mice were divided into groups of control, Rel vaccine, and MIP-3α-Rel vaccine, with all given INH. Mice were harvested at 10 weeks post-treatment initiation, and homogenized lungs were plated in serial dilutions to calculate colony-forming unit (CFU) infectious burden data (Fig. 1A)^10^. Our previous study found that Rel vaccination increased the bactericidal activity of INH, and MIP-3α-Rel vaccination further increased its activity, leading to significantly reduced lung CFU burden^10^. Figure 1B shows that upon secondary analysis, it was clear that the therapeutic vaccines had a sex bias in their efficacy. Stratifying the data by sex and analyzing by Two-Way ANOVA with treatment and sex as the two independent variables, no difference in CFU was observed between the sexes in mice receiving INH alone. Females in the INH + Rel group had 57% fewer CFU compared to their male counterparts (*p* = 0.0039), and for the INH + MIP-3α-Rel group, females had 74% fewer CFU compared to their male counterparts (*p* < 0.0001). Additionally, the interaction term was significant (*p* = 0.0041), suggesting that therapy modality had differences in efficacy depending on the sex of the mouse. Importantly, despite the sex bias, MIP-3α fusion enhanced the efficacy of the Rel vaccine in both sexes. These data showed the sex bias of our vaccine model and provided evidence that inclusion of MIP-3α in the vaccine construct enhanced the overall efficacy in both sexes but also heightened the female efficacy bias. Since MIP-3α is known to target CCR6+ iDCs and Langerhans cells^11–17^, these observations led to our hypothesis that sex differences in APC function, specifically differences in targeting vaccine antigen to DCs, are likely contributing to the observed female bias.

### EαGFP and MIP-3α-EαGFP model system and validation

**Fig. 2:**
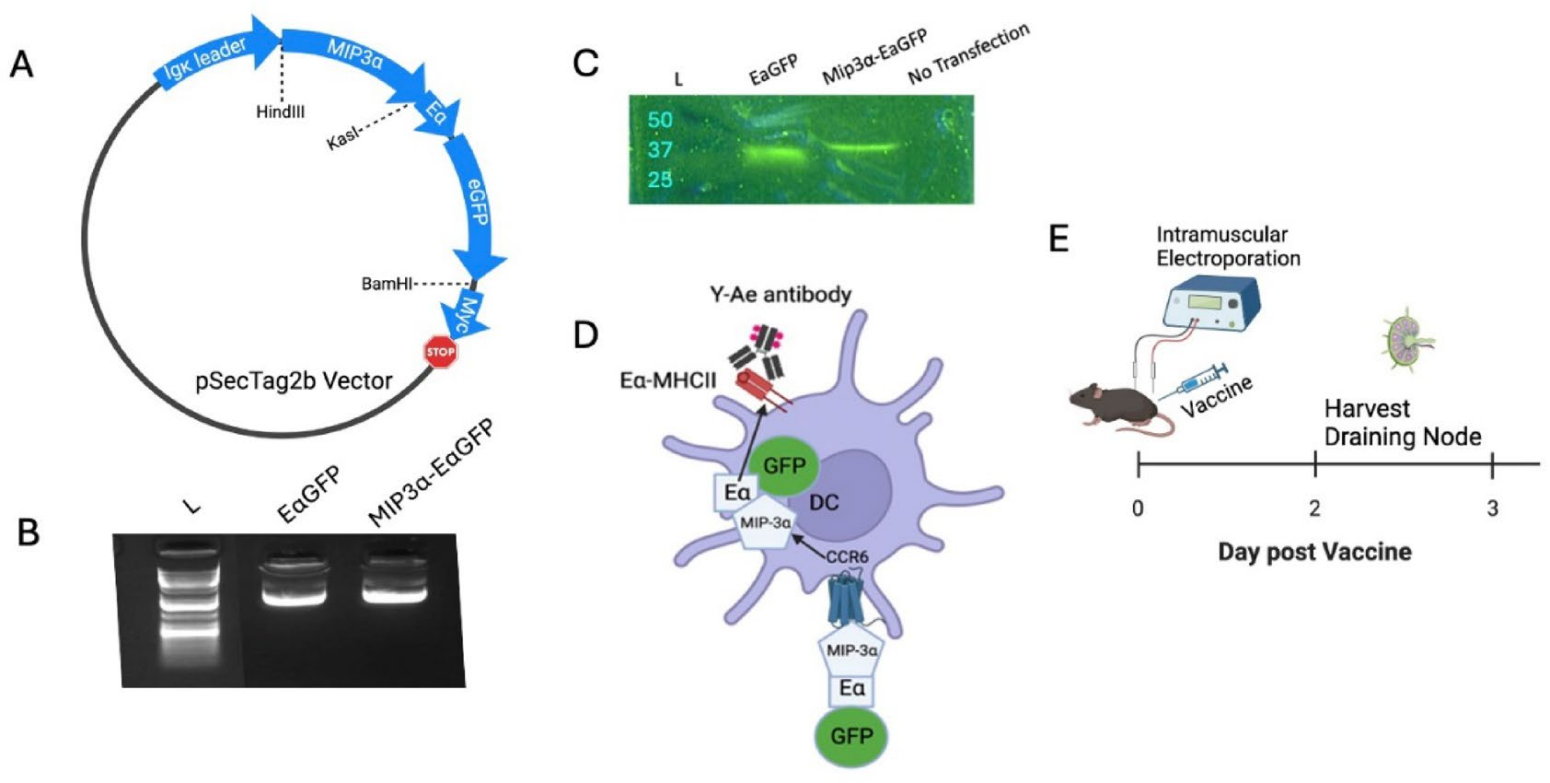
MIP-3α-EαGFP Model. (**A**) Map of expressed region of vaccine plasmid with regions and cloning sites labeled. (**B**) Confirmations of transfection-grade plasmid preps to show DNA purity and supercoiling (**C**) EαGFP and MIP-3α-EαGFP vaccines verified in vitro by transfection of HEK293T-cells. Cell media was analyzed by semi-denaturing gel followed by blotting and GFP visualization under UV excitation and a green filter (**D**) Model of system. Secreted vaccine protein will be targeted to iDCs via interaction between MIP-3α and CCR6. Internalization can be tracked by GFP and antigen presentation by Y-Ae antibody designed to interact with I-Ab MHC-II loaded with Eα peptide. (**E**) Schedule of manuscript experiments: Vaccines administered into mouse gastrocnemius muscle with electroporation. Draining popliteal nodes were harvested two to three days later. Illustrations made using Biorender.com.

**Fig. 3:**
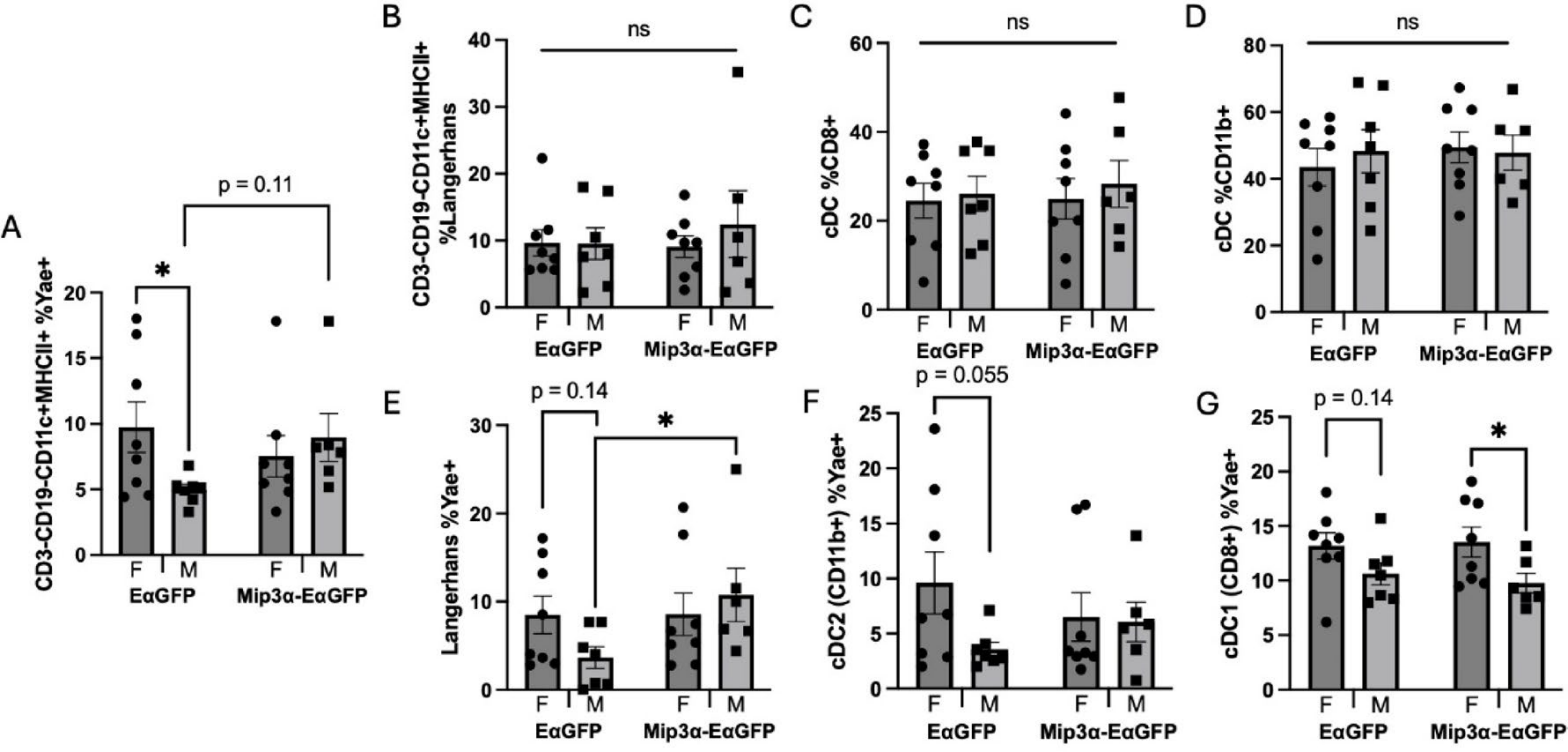
Sex and MIP-3α differences of antigen presentation. Eα peptide, inherent in our reporter vaccines as described in Fig. 2, has a known antibody (Y-Ae) that binds to the processed peptide in complex with I-A^b^ MHC-II on C57Bl/6 mouse APCs, allowing for identification of processed vaccine antigen. Two days post immunization, draining popliteal nodes were harvested, processed, and analyzed by flow cytometry. (**A**) Antigen presenting cells (APCs) as defined by CD3-CD19-CD11c+ MHCII+ shown as percentage positive for Y-Ae signal. Panels B-D show overall lymph node populations of APCs, including Langerhans cells (**B**); CD207+F4/80+), and cDCs (CD207-F4/80−) divided into CD8+ (**C**) and CD11b+ (**D**) populations. Panels (**E**–**G**) define the Y-Ae+ proportions of those APC subsets. Data are representative of two independent experiments, n = 6–8. *p < 0.05, ns, not significant, trending *p*-values labeled.

**Fig. 4:**
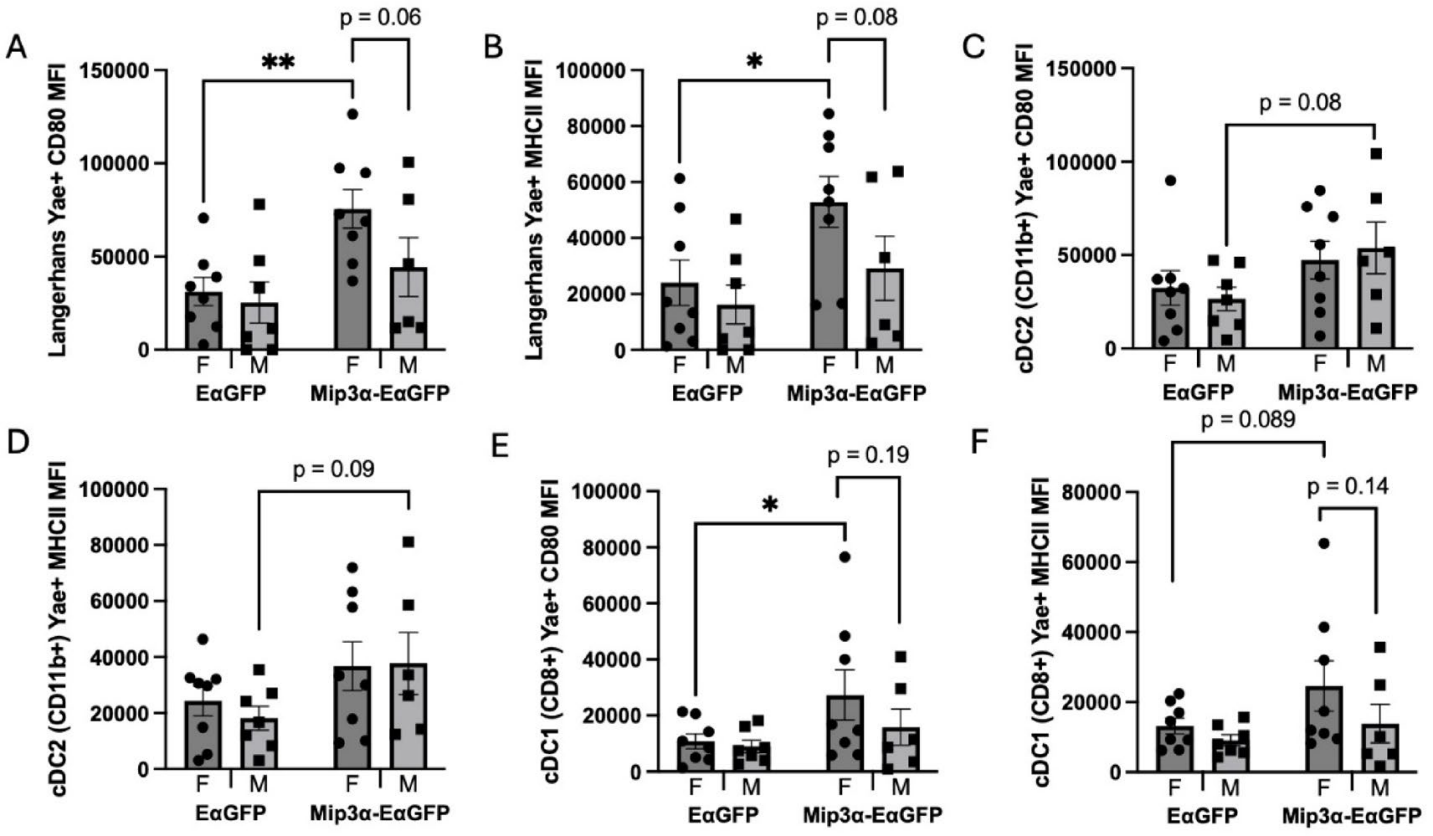
Sex and MIP-3α differences of APC activation within Y-Ae positive populations. Two days post immunization, draining popliteal nodes were harvested, processed, and analyzed by flow cytometry. Langerhans (**A**, **B**), CD11b+ cDC (**C**, **D**), and CD8+ cDC (**E**, **F**) cell populations were analyzed for surface expression levels of CD80 (**A**, **C**, **E**) and MHCII (**B**, **D**, **F**) by comparing mean fluorescence intensity (MFI) measures. Data are representative of two independent experiments, n = 6–8. *p < 0.05, **p < 0.01, trending p-values labeled.

**Fig. 5:**
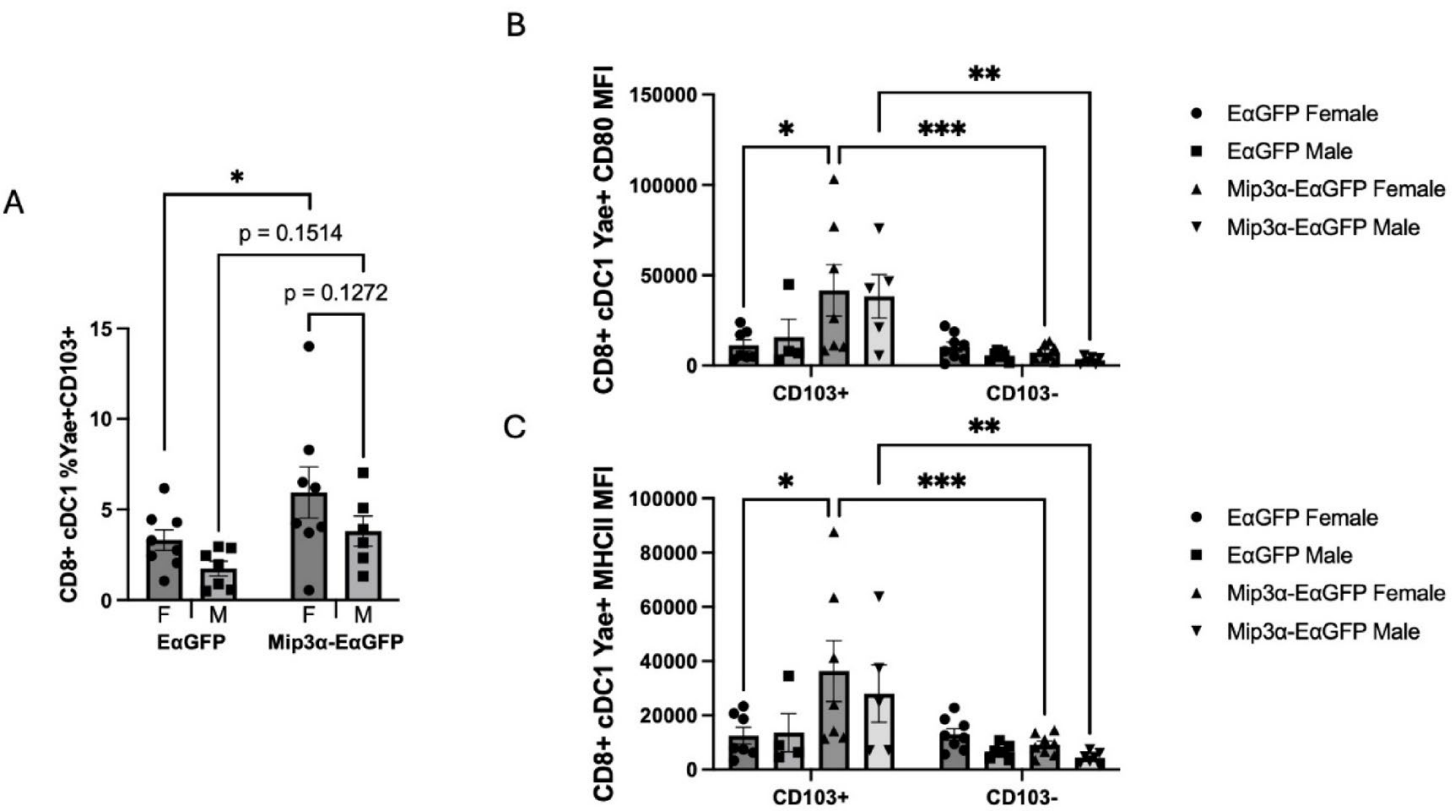
Sex and MIP-3α differences of the CD8+ cDC population positive for both Y-Ae and CD103. Two days post immunization, draining popliteal nodes were harvested, processed, and analyzed by flow cytometry. (**A**) Proportion of CD8+ cDC cells positive for both Y-Ae and CD103. (**B**) CD80 and (**C**) MHCII MFI measurements of the CD8+ cDC population positive for Y-Ae stratified by CD103 positivity. Data are representative of two independent experiments, n = 6–8. *p < 0.05, **p < 0.01, ***p < 0.001, trending p-values labeled.

**Fig. 6:**
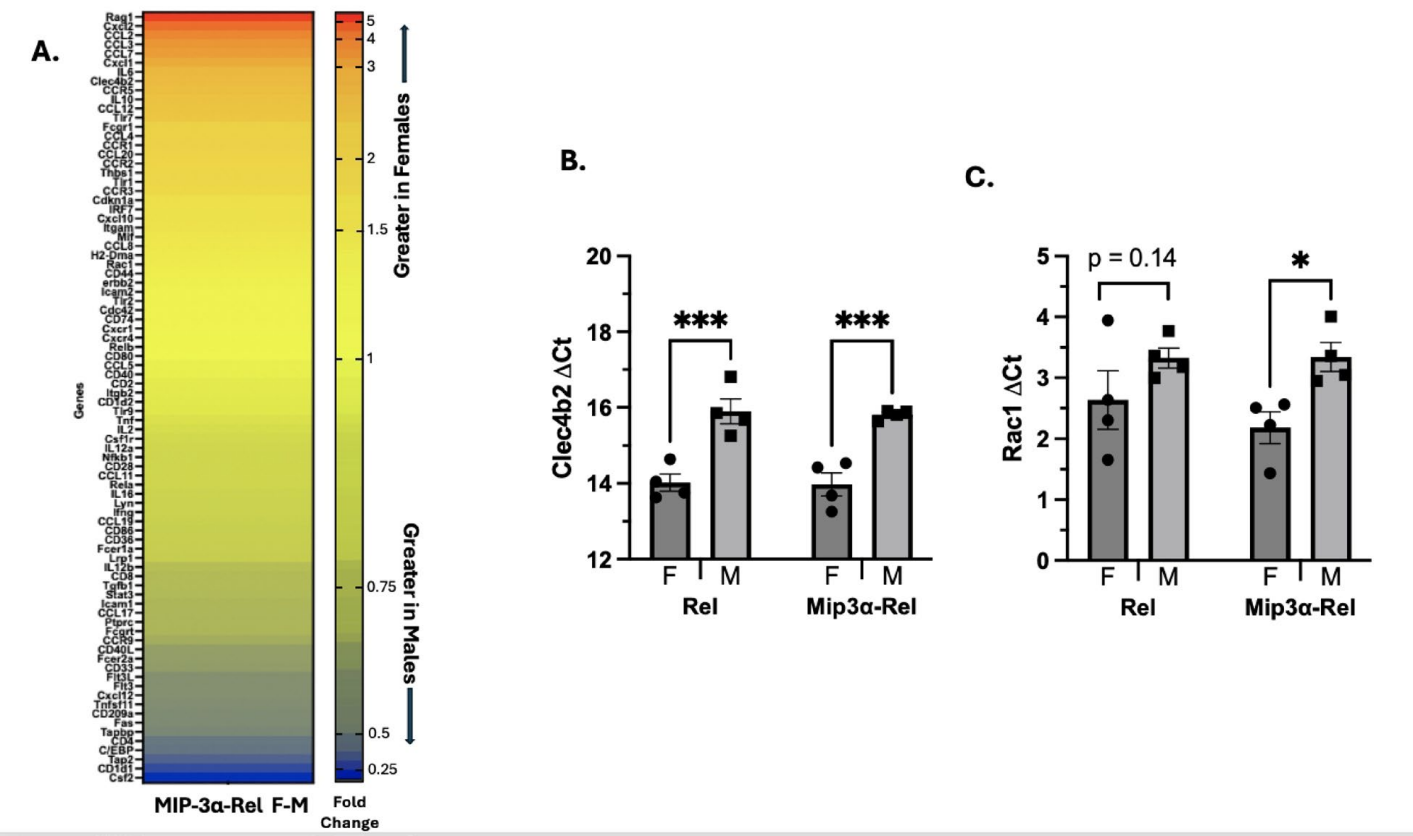
qRT-PCR analyses of differentially expressed genes between sexes. (**A**) Mice were immunized with MIP-3α-Rel and popliteal nodes were harvested at day 3, RNA was extracted, and qRT-PCR was performed by comparing pooled samples of MIP-3α-Rel vaccinated females to MIP-3α-Rel vaccinated males utilizing the Mouse Dendritic and Antigen Presenting Cell qRT-PCR Array. Data are presented as a heat map of the the calculated fold change (2^−ΔΔCt^) with higher values representing more relative transcription in female mice. (**B**, **C**) Mice were immunized with MIP-3α-Rel or Rel and popliteal nodes were harvested at day 2, RNA was extracted, and qRT-PCR was performed analyzing the specific genes (**B**) Clec4b2 and (**C**) Rac1, with data represented as ΔCt. *p < 0.05, ***p < 0.001, trending *p*-values labeled.

To detect and track which cells take up and present vaccine antigen, our laboratory created EαGFP and MIP-3α-EαGFP reporter vaccines (Fig. 2A), with verified plasmid purity (Fig. 2B) and in vitro transfection ability (Fig. 2C), with uncropped images in Supplemental Fig. 2. The EαGFP vaccine functions as a DC reporter^26,27^ by providing two mechanisms for tracking: GFP as a visible cue for phagocytosed protein and the Eα peptide, which has a known antibody (Y-Ae) that binds to the processed peptide in complex with I-A^b^ MHC-II on C57Bl/6 mouse APCs, allowing for identification of processed vaccine antigen (Fig. 2D). Mice were vaccinated with the EαGFP or MIP-3α-EαGFP reporter vaccine (Figs. 3, 4, 5) or with Rel or MIP-3α-Rel (Fig. 6). At 48 and 72 h post-vaccination as indicated, popliteal nodes were harvested (Fig. 2E) and either processed into single-cell suspensions for flow cytometry or had RNA extracted for qRT-PCR.

### Sex and MIP-3α differences in proportion of APC’s presenting antigen

An initial hypothesis was that MIP-3α was enhancing overall vaccine antigen presentation, which is known to have a female bias in other model systems^22,25^. In Fig. 3, both sex and vaccine type differences are shown in APC subtype and the surface distribution of Y-Ae-labeled vaccine antigen distribution. The gating strategy is shown in Supplemental Figs. 3 and 4. All presented data were selected for immune cells by forward scatter (FSC) vs side scatter (SSC), single cells, alive cells, CD3-, CD19-, CD11c+, and MHC-II+, defining APCs generally. Confirming the female antigen presentation bias, we found a significant sex difference in the percentages of Y-Ae+ APCs amongst EαGFP groups, with females averaging about twice that of males (*p* = 0.042). Unexpectedly, the MIP-3α-EαGFP groups showed equivalent APC presentation levels in both sexes. When comparing the same sex across the vaccine types, the results show a trending difference in males vaccinated with the construct containing MIP-3α (mean 74.2% higher Y-Ae signal; *p* = 0.11) relative to those vaccinated only with EαGFP (Fig. 3A). Overall, APC antigen presentation in the standard vaccine model showed a significant female bias, as hypothesized, but the MIP-3α-containing vaccine clearly displayed different dynamics.

We next analyzed the overall cell infiltrate to see if MIP-3α-EαGFP immunized animals had overall shifts in APCs trafficking to the popliteal lymph nodes. When comparing APC subtypes (Fig. 3B–D), there are no significant differences in sex or vaccination type in the percentage of Langerhans cells (Langerin+, F4/80+), type 1 conventional DCs (cDC1s; Langerin-, F4/80−, CD8+), or type 2 conventional DCs (cDC2s; Langerin−, F4/80−, CD11b+). There were also no significant differences in the percentage of alive T-cells, B-cells, or GFP^+^ cells by sex or vaccine type that could confound the results (Supplementary Fig. 5). From these data, we concluded that the overall cell composition of the node remained consistent independent of vaccine formulation and sex.

Next, to test the hypothesis that there may be differences in antigen uptake across the specific APC subtypes, we analyzed which cells were positive for reporter vaccine antigen. Among the groups receiving the EαGFP construct, females trended higher than males across all three subtypes positive for Y-Ae: Langerhans cells (2.32-fold higher; *p* = 0.14; Fig. 3E), cDC2s (2.68-fold higher; *p* = 0.055; Fig. 3F), and cDC1s (23.8% higher; *p* = 0.14; Fig. 3G). In contrast to these results, the MIP-3α-EαGFP groups showed equivalence in Y-Ae positivity across sexes in Langerhans and cDC2 cells (Fig. 3E,F), but importantly showed a significant sex difference in cDC1 cells, with females averaging 38.5% higher than males (*p* = 0.04; Fig. 3G). When comparing within the same sex across vaccination groups, MIP-3α-EαGFP males had a Y-Ae positivity rate in Langerhans cells that was 2.94 times higher that of males receiving Eα-GFP (*p* = 0.045; Fig. 3E).

Overall, the APC subtypes (Fig. 3E–G) mimic the antigen presentation data (Fig. 3A) for the EαGFP vaccine, with females trending higher in each, showing that the female bias in antigen presentation at baseline was not skewed to any specific subtype of APC. In the MIP-3α-EαGFP group there is a significant female bias for cDC1s to uptake vaccine antigen and present it to T-cells in the lymph node, but not in the other subtypes. Interestingly, the only differences found across vaccination groups within the same sex was an increase in antigen presentation in Langerhans cells in male mice. These data support our theory that the sex differences observed in response to vaccination is in part due to different preferences for APC subtypes. Notably, though, there is not a consistent difference in the same sex analyses across vaccine types, so these data alone can’t explain the role played by MIP-3α in the vaccine enhancement.

### Sex and MIP-3α differences in APC activation levels

In addition to studying the ex vivo antigen presentation proportions across APC subtypes, we also examined activation markers of the APCs presenting Y-Ae to determine if there were sex differences and/or MIP-3α differences as a result of vaccination. Since MIP-3α enhances vaccine efficacy and increases endpoint T-cell responses^11–17^, which could not be fully explained by increased numbers of antigen loaded-APCs in the draining node (Fig. 3), we hypothesized that MIP-3α could increase DC activation, potentially leading to a more robust T-cell response. Therefore, we examined the differences in surface expression of CD80 and MHC-II in Langerhans cells, cDC2 (CD11b+) DCs and cDC1 (CD8+) DCs presenting vaccine peptide by analyzing mean fluorescence intensity (MFI) of flow cytometry results.

Figure 4 highlights that EαGFP vaccinated animals did not exhibit any sex-biases in activation levels across all subtypes of Y-Ae positive APCs. In contrast, MIP-3α-EαGFP vaccinated female mice exhibited trends in sex biases of APC activation in Langerhans and cDC1 cells but not cDC2 cells. Female MIP-3α-EαGFP mice had Y-Ae + Langerhans cells with 70.4% higher CD80 (Fig. 4A, p = 0.06) and 81.2% higher MHC-II (Fig. 4B, p = 0.08) surface abundance compared to males. Similarly, cDC1 cells show MIP-3α-EαGFP females averaging 72.6% higher CD80 (*p* = 0.19; Fig. 4E) and 77.9% higher MHC-II levels than males (*p* = 0.14; Fig. 4F).

Critically, Fig. 4 does show that the addition of MIP-3α to the vaccine enhances activation levels of all vaccine-specific APC subtypes to varying degrees. In Y-Ae+ Langerhans cells, female MIP-3α-EαGFP mice have significantly increased levels of CD80 by 2.42-fold (*p* = 0.0061; Fig. 4A) and MHC-II by 2.2-fold (*p* = 0.023; Fig. 4B) compared to female recipients of EαGFP. In Y-Ae+ cDC2 cells, male MIP-3α-EαGFP mice have trending higher levels of CD80 (twofold; *p* = 0.08; Fig. 4C) and MHC-II (2.1-fold; *p* = 0.09; Fig. 4D) compared to male EαGFP mice. Finally, in Y-Ae+ cDC1 cells, female MIP-3α-EαGFP mice have significantly higher levels of CD80 (2.5-fold; *p* = 0.049; Fig. 4E) and trending higher levels of MHC-II (86.7%; *p* = 0.089; Fig. 4F) compared to female EαGFP mice.

Overall, the findings in Fig. 4 support the hypothesis that the enhanced adjunctive bactericidal activity afforded by the MIP-3α fusion (Fig. 1) is likely not driven simply by enhanced antigen presentation, but at least in part by enhanced activation of APCs, which are better able to productively present antigen to T-cells. Further, expression of APC activation markers was most robust in female mice, potentially explaining the observed sex bias of the therapeutic efficacy of MIP-3α-Rel.

### Cross-presenting CD8+CD103+DCs

We found that cDC1 DCs from MIP-3α-EαGFP vaccinated mice had a female bias in both vaccine antigen presentation (Fig. 3G) and cell surface activation marker levels (Fig. 4E,F). We also found that cDC1 DCs from MIP-3α-EαGFP vaccinated mice had higher activation levels than EαGFP vaccinated mice (Fig. 4E,F). These data provide evidence as to the import of this cell type and led us to investigate these cells further to determine the proportion that were lymphoid resident (CD103-) or that had trafficked from the vaccine site (CD103 +). We found that MIP-3α enhances the number of Y-Ae+ and CD103+ positive cells, with MIP-3α-EαGFP females averaging 78.9% higher than EαGFP females (*p* = 0.046) and MIP-3α-EαGFP males averaging 2.2 times higher than EαGFP males (*p* = 0.15) (Fig. 5A). There is also a trending sex difference in the MIP-3α-EαGFP group with females averaging 56.3% higher than males (*p* = 0.13). This provides evidence that a greater proportion of the Y-Ae+ cDC1 DCs observed in Fig. 3F are from the CD8+ CD103+ subtype in the MIP-3α-EαGFP group as compared to the EαGFP group, while maintaining similar sex difference proportions.

We then stratified the activation signal data from the cDC1 Y-Ae + population by CD103 positivity. CD80 (Fig. 5B) and MHCII (Fig. 5C) MFI levels show a clear phenotype: the majority of the enhanced activation signal observed in cDC1 cells (Fig. 4E,F) are attributable to the CD8+ CD103+ subset, which are almost exclusively present in the MIP-3α-EαGFP groups. A comparison of the activation levels across the vaccine groups within each sex revealed that MIP-3α-EαGFP females had significantly higher CD80 (3.7-fold; *p* = 0.011) and MHCII (2.9-fold; *p* = 0.014) MFI than EαGFP females. Although males showed a similar phenotype, no statistical difference was observed, in large part due to a loss of statistical power from several samples in the EαGFP males being excluded due to too few CD103+ cDC1s to analyze by MFI. Interestingly, activation levels did not show any sex bias. These findings in part explain why female mice receiving MIP-3α-EαGFP had the most robust response to therapy (Fig. 1), since they had the highest proportions of CD8+ CD103+ DCs (Fig. 5A), which are highly activated by MIP-3α (Fig. 5B,C).

### Analysis of total lymphoid RNA

To determine which genes might be contributing to the sex difference phenotype, mice were vaccinated with either Rel or MIP-3α-Rel, and then RNA was isolated from mouse popliteal nodes 3 days post-vaccination and processed for the RT-qPCR array. Figure 6A outlines the fold difference between female and male mice after MIP-3α-Rel vaccination (ΔΔCt: Female ΔCt – Male ΔCt; fold difference: 2^-ΔΔCt^). Females were found to express Rag1, CCL12, CCL2, CCL20, CCL3, CCL4, CCL7, CCR1, CCR5, CXCL1, Clec4b2, CXCL2, FCGR1, IL-10, IL-6, and TLR7 at a > twofold higher level than males. Males expressed CD1d1, CD209a, CD4, C/EPB, CSF2, Fas, TAP2, and TAPBP at a nearly twofold higher level than females. Based on the sex difference data from the RT-qPCR array, the previous flow data, and the literature, Rac1 was selected to repeat qRT-PCR analysis due to its effects on DC motility^29^, which could impact DC infiltration at the site of vaccination. Clec4b2 was also selected to analyze as it impacts CD8 + T-cell cross-presenting^30^, which is highly prevalent in cDC1 DC subtypes^31^. Figure 6B,C outline the sex and MIP-3α differences in ΔCt values for these genes. We observed a significant difference in Clec4b2 ΔCt between the sexes for the Rel vaccinated mice (*p* = 0.0002) and the MIP-3α-Rel vaccinated mice (*p* = 0.0003). There was no difference seen between the Rel vaccine group and the MIP-3α-Rel vaccine group (Fig. 6B). This supports our findings that female mice have greater numbers of cDC1 DCs that can cross-present antigen. We also found there is a significant difference in Rac1 ΔCt between the sexes for the MIP-3α-Rel vaccine group (*p* = 0.021). A trending sex difference is also present in the Rel group (*p* = 0.14; Fig. 6C). This also indicates that females may have increased motility, leading to more infiltrating cDC1 DCs at the site of vaccination.

## Discussion

Utilizing the EαGFP reporter vaccine is a powerful tool to characterize the APCs that are actively taking up and presenting vaccine antigen, including the effects vaccine formulations and sex have on these APCs in the days following vaccination. As described in Fig. 2D, the Eα peptide, when presented by I-A^b^ MHC-II molecules in C57BL/6 mice, can be analyzed with the antibody Y-Ae in flow cytometric assays to determine Eα presentation for different APC types. Based on previous results in in vitro DC models with MIP-3α fusion vaccines, it has been shown that targeting with CCR6 was able to induce successful cross-presentation of antigens into both MHC-I and MHC-II pathways^15–17^. However, this study is the first to analyze the effects of MIP-3α on APCs ex vivo to assay the real time antigen presentation capabilities of different types of APCs post-vaccination. This is also the first study to assess how MIP-3α fusion to a vaccine antigen affects differences in the sexes in regard to both microbiological endpoints and early-stage APC activity. Additionally, this is the first study to our knowledge to assess sex differences in vaccine uptake utilizing the Eα peptide – Y-Ae antibody tracking system.

In the literature, females have shown to have overall more active immune responses compared to males. Specifically, female APCs are more efficient in phagocytosis, antigen presentation, and trafficking to the lymph node than males^21–23^. Studies have also found that estrogens can enhance DC development and function^25^, and estradiol can increase IL-6 and MCP-1 (CCL2) production by iDCs, enhance DC migration towards chemokines in the lymph node, and increase DC ability to stimulate T-cells without fundamentally altering the DCs^32^, Based on previous studies, we hypothesized that females would show greater levels of antigen presentation and that MIP-3α presence in the vaccine would enhance presentation in DCs coming from the vaccination site and able to cross-present antigens. Overall, our data support this hypothesis with some further intriguing findings.

When comparing sex and MIP-3α differences for vaccine antigen uptake in different DC subsets, we found that MIP-3α increased Y-Ae + DC infiltration from the periphery, with MIP-3α males having the highest percentage of Langerhans cells and MIP-3α females the highest percentage of CD8 + CD103 + dermal DCs (Fig. 3, 5). We also observed no differences in general numbers of APCs, T-cells, B-cells or GFP by sex or vaccine type that could confound the results (Fig. 3B–D; Supplementary Fig. 5). CD8+ CD103− DCs are non-migratory cells that reside in the lymph node^33^ and CD8+ CD103+ are dermal DCs with high migratory potential that, when presented with antigen, travel to the draining lymph node^34^. Both cDC1 subtypes (CD8+ and CD8+ CD103+) are known to effectively cross-present antigen to CD8+ T-cells upon activation^31^. Further, CD8+ CD103+ DCs have been found to be essential in the phenomenon of DC cross-dressing, which is especially important in DNA vaccinations^31^. In the literature, there are known sex differences in these DC subsets and in CD8+ T-cell activation. A recent study found that DCs from female mice were better at taking up FITC injected into the skin than male counterparts^35^, which is consistent with our antigen presentation data. Additionally, while male mice typically have increased numbers of CD8+ T-cells, female mice have higher numbers of activated CD8+ T-cells in different models^18,36–38^, which could result from increased cross presentation from cDC1 (CD8+ and CD8+ CD103+) DCs. Previous studies in an asthma model have also found that MHC II expression and CD103+ DC antigen uptake was higher in females than males^23^, providing evidence for why females have more cDC1 (CD8+ CD103+) Y-Ae+ DCs compared to males. These factors contribute to increased CD8+ T-cell cross-presentation/dressing and activation, and a more favorable overall immune response in female mice.

In addition to sex differences, we also saw MIP-3α differences in activation due to vaccination (Fig. 4). In Y-Ae+ Langerhans cells, cDC1s (CD8+), and cDC2s we found MIP-3α groups had the highest CD80 and MHC II surface expression when compared to the EαGFP groups. These data indicate that regardless of APC subtype, mice immunized with MIP-3α-fusion vaccines have APCs with greater surface levels of activation markers. We also observed a female sex bias in activation in the Langerhans cells and cDC1 (CD8+) DCs. A majority of the CD80 and MHC II surface expression observed in the cDC1 DCs was from CD8+ CD103+ DCs, which are greatly increased in animals vaccinated with the MIP-3α-fusion vaccine (Fig. 5). Another study found that female mice had more mature CD103+ cDC1 cells in the skin than male mice^35^, which could be contributing to why we may see more nodal trafficking and activation by this subtype. Further details into the mechanisms of activation, cross-presenting or dressing, and nodal trafficking will be the focus of future studies.

These findings are further supported by our qRT-PCR panel in Fig. 6A. Many of the genes upregulated in MIP-3α females are chemokines involved in immune cell motility and attracting cells to the node, such as CCL12, CCL2, CCL3, CCL7, CCR1, CCR5, and CXCL1^39,40^. Figure 6C shows that females have enhanced Rac1 expression, trending in the Rel group and significant in the MIP-3α-Rel group. Rac1 is a Rho GTPase that has many functions including actin cytoskeleton rearrangement^29^ which may lead to increased DC motility. This is also consistent with the literature as studies have found that estradiol is essential for DC migration^32^. Increased cell motility in females could result in more periphery DCs moving to the site of antigen exposure to process and uptake vaccine antigen. High levels of chemokines could also increase dermal DC migration to bring vaccine antigen to the draining lymph nodes, contributing to more CD8+ T-cell cross-presentation and resulting in an improved immune response to vaccination.

Figure 6A also highlights that many of the genes upregulated in female mice receiving the MIP-3α construct are associated with CD8+ T-cell recruitment and cross-presentation. In the literature, CCR6/CCL20 recruits DCs that are responsible for CD8+ T-cell cross-presenting^41^ and CCL3 can enhance CD8+ T-cell infiltration through CD103+ DC recruitment^42^. Studies have also shown after vaccination, naïve CD8+ T-cells in the draining lymph node secrete CCR5 which recruits immune cells that secrete CCL3 and CCL4, resulting in increased memory CD8+ T-cells in the nodes^43^. This is further supported in Fig. 6B where Clec4b2, or DCAR1, is significantly expressed more in females than males in both groups. Clec4b2 is found on the subset of cDC1 (CD8+) DCs and is involved in T-cell cross-presenting^30^. Coupled with the increased levels of Y-Ae+ cDC1(CD8+ and CD8+ CD103+) DCs, these genes could also contribute to the enhanced cross-presenting of CD8+ T-cells, thus enhanced immune response in females. More detailed analysis of T-cell response induction will be the subject of future work.

The genes that are upregulated in males receiving the MIP-3α construct include CD209a (DC-SIGN), especially important in monocyte-derived DC-driven T-cell responses^44^, and multiple genes that signal or induce DC maturation, such as C/EPB, Fas, and Tap2^45–47^. This provides further evidence that MIP-3α is associated with sex biases in terms of which APC types are targeted, and further studies will need to confirm the role of monocyte-derived DCs in this system. These data also suggest the female APCs likely matured before this time point, considering the higher surface levels of activation markers observed in the female MIP-3α group (Figs. 4, 5) combined with this genetic shift towards males with maturation gene expression (Fig. 6A). The location and temporal dynamics of cellular activation will also be analyzed in greater detail in future studies.

## Conclusions

Overall, our studies show that therapeutic vaccination with the *M. tuberculosis* antigen Rel_Mtb_ enhances the bacterial clearance by antibiotics with a female bias, and that fusion of the antigen to MIP-3α enhances both the overall response and the sex bias. The data presented here support the hypotheses that fusing MIP-3α to the antigen leads to better targeting of APCs at the vaccination site, such as Langerhans and CD103+ dermal DCs, and that female APCs have better antigen presentation capacity than males, including cross-presenting DCs. Interestingly, we found that the APCs from mice immunized with MIP-3α-fusion led to increased APC activation. We hypothesize the enhancement of therapeutic efficacy is in part due to the intriguing increase in APC activation markers primarily in the MIP-3α-antigen immunized mice combined with a female bias of cDC1 (CD8+ and CD8+ CD103+) DC vaccine antigen presentation, resulting in highly activated DCs that can cross-present to CD8+  T cells, prompting them to elicit a strong immune response. Future investigations will track nascent T-cell responses in the days and weeks after vaccination and will also assess the type 2 innate lymphoid cell (ILC2) population as a recent study suggests that they play a major role in sex differences regarding DC accumulation and activation and are negatively regulated by androgens^35^. More research studying the mechanisms involved in the sex differences observed as a result of vaccination, such as direct and indirect hormonal influences as well as sex-linked genetic influences, will be pivotal to better understand the sex biases of vaccine systems.

## Electronic supplementary material

Below is the link to the electronic supplementary material.

Supplementary Material 5

Supplementary Material 2

Supplementary Material 6

Supplementary Material 7

Supplementary Material 8

Supplementary Material 4

Supplementary Material 1

Supplementary Material 3

Supplementary Material 9

## Data Availability

Presented data are provided in the submission supplement. Any other data or materials are available upon reasonable request.
