# Supplementary figures and images for: MIP-3α-antigen fusion DNA vaccine enhances sex differences in tuberculosis model and alters dendritic cell activity early post vaccination
Source: Sci Rep. 2025 Jul 1;15:22264. doi: 10.1038/s41598-025-06532-6 (PMC12218986; doi:10.1038/s41598-025-06532-6)

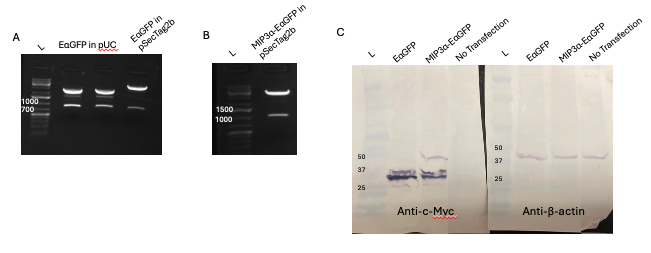

Supplement: Supplementary file 1 — Supplementary Material 5 [file 41598_2025_6532_MOESM1_ESM.jpg]

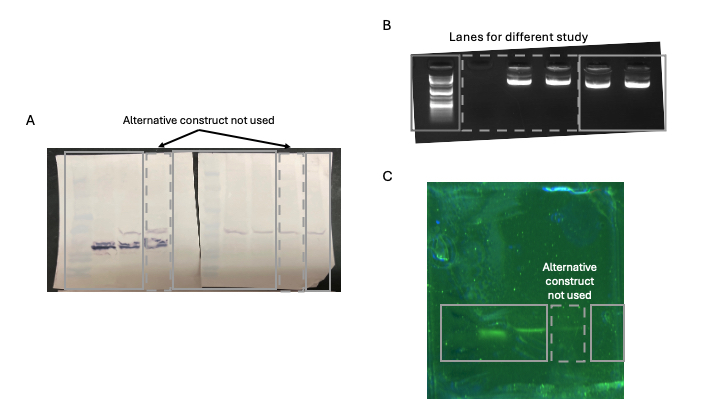

Supplement: Supplementary file 2 — Supplementary Material 2 [file 41598_2025_6532_MOESM2_ESM.jpeg]

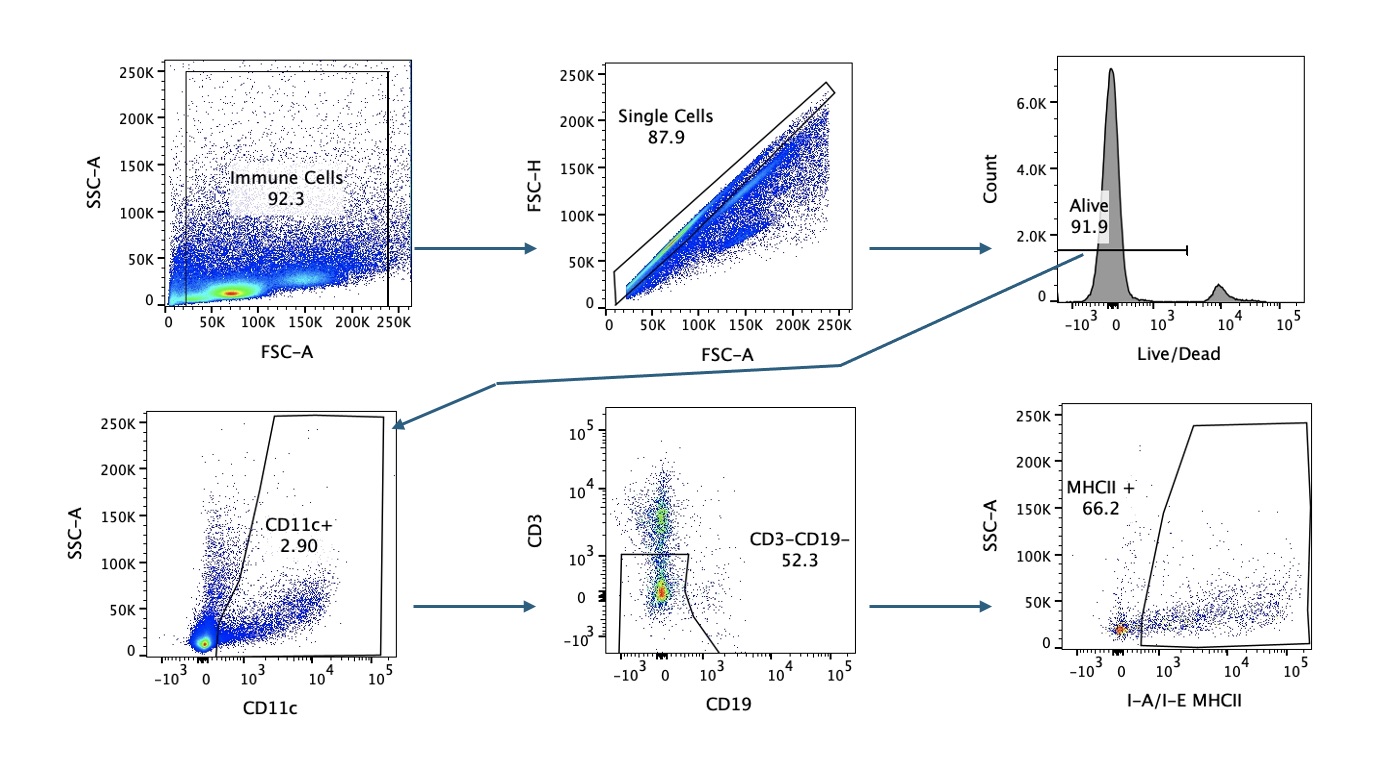

Supplement: Supplementary file 3 — Supplementary Material 6 [file 41598_2025_6532_MOESM3_ESM.jpeg]

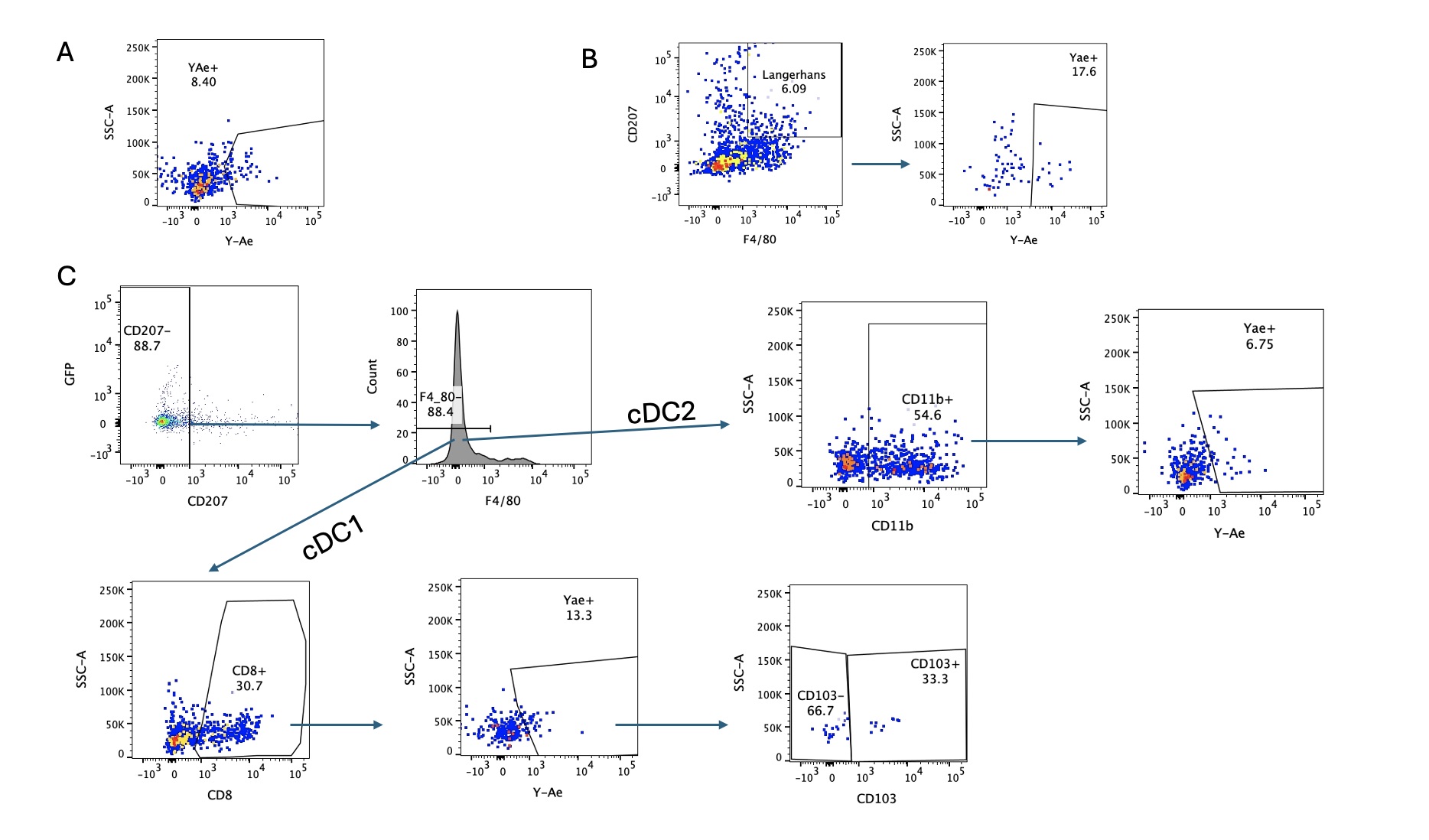

Supplement: Supplementary file 4 — Supplementary Material 7 [file 41598_2025_6532_MOESM4_ESM.jpeg]

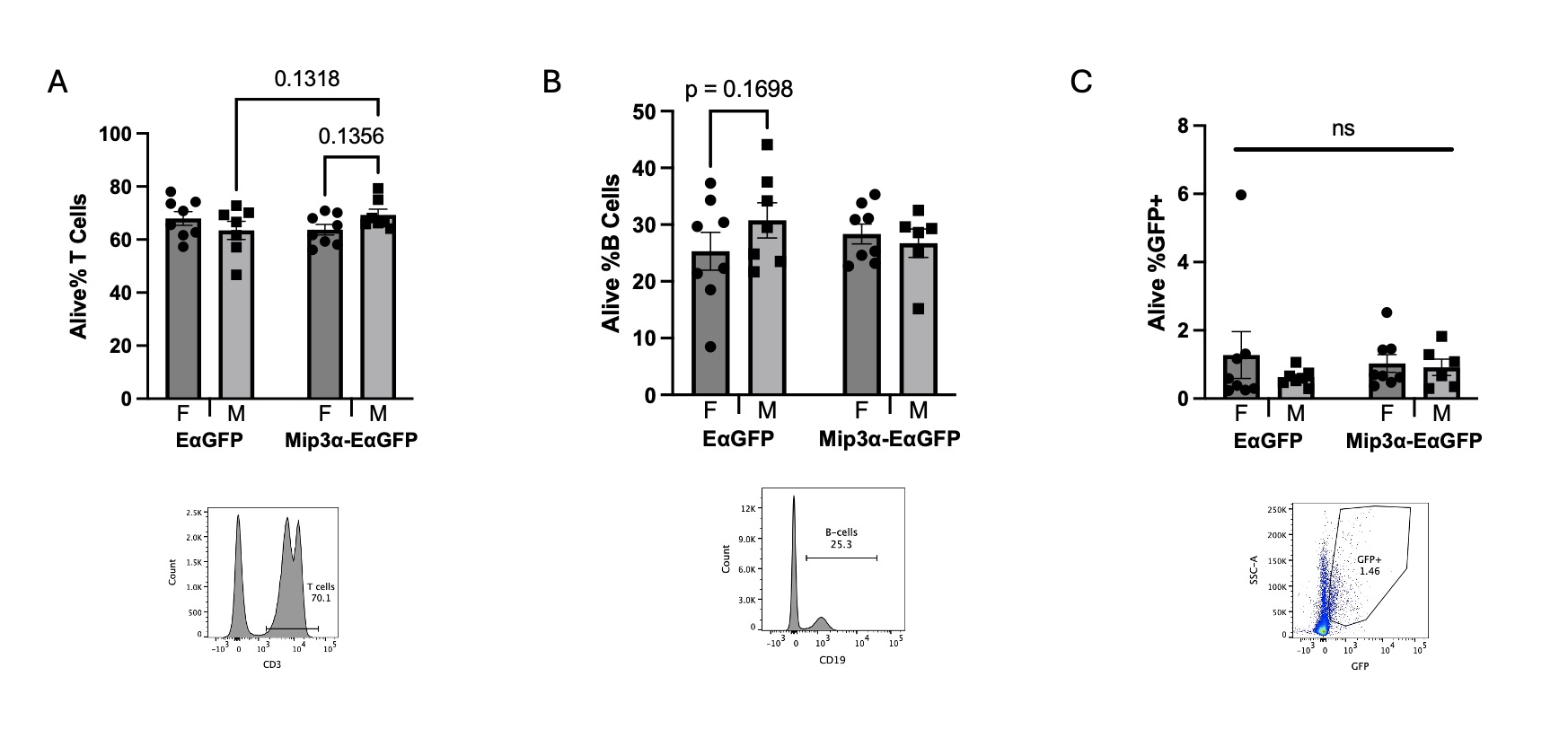

Supplement: Supplementary file 5 — Supplementary Material 8 [file 41598_2025_6532_MOESM5_ESM.jpeg]

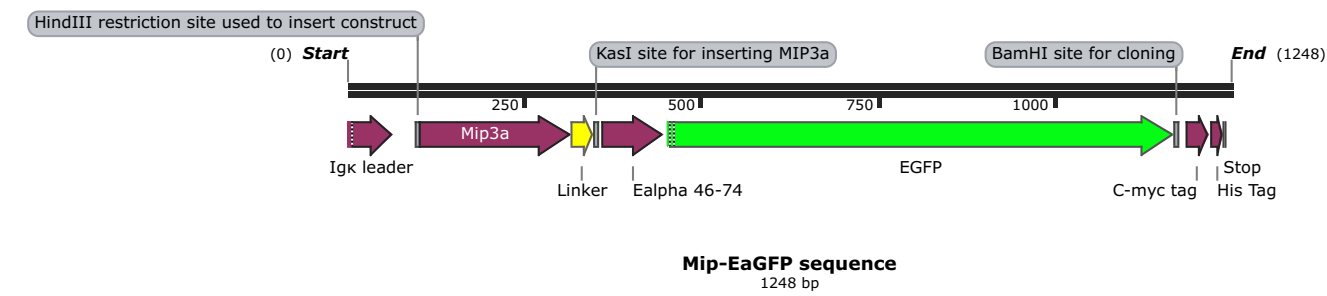

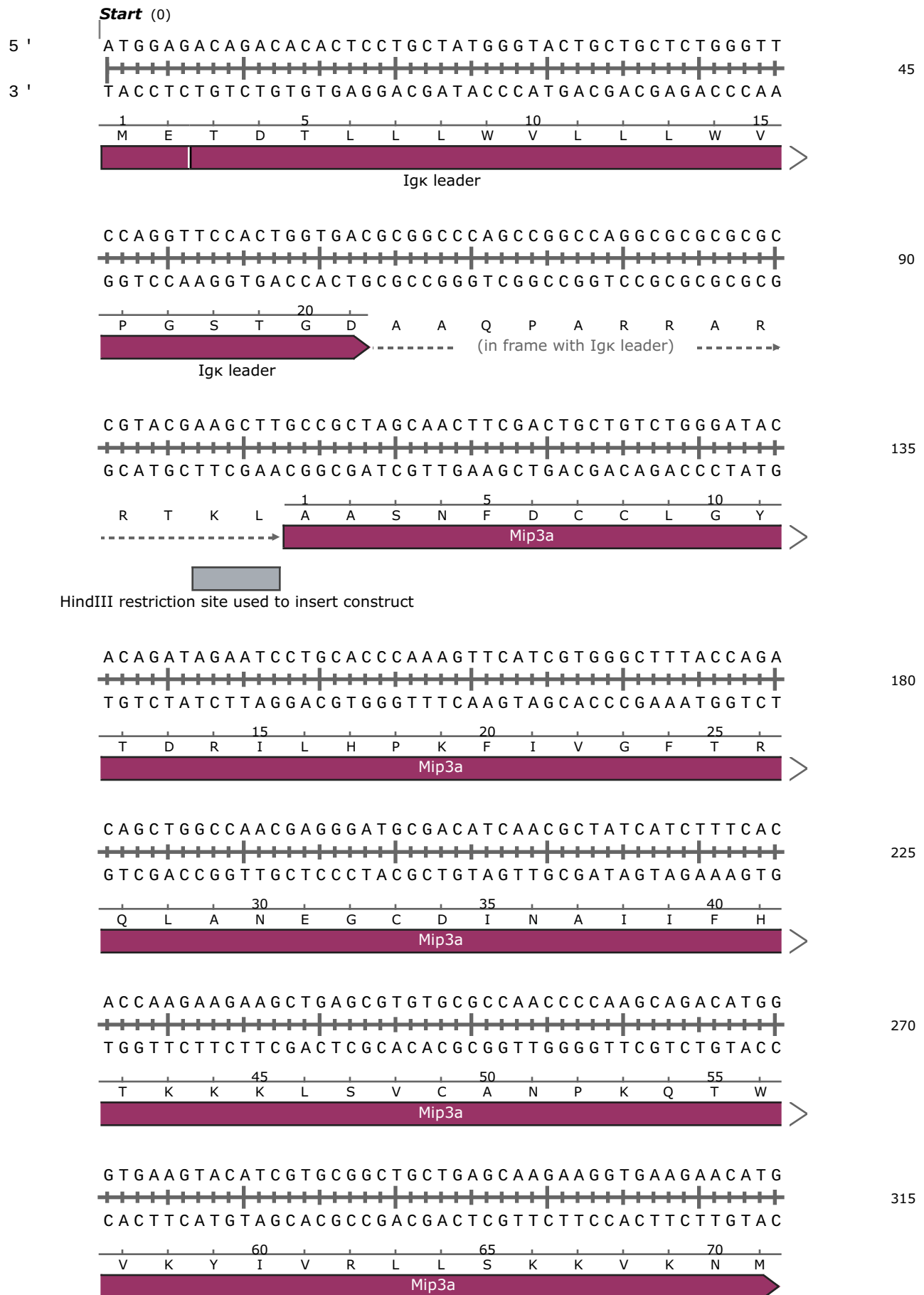

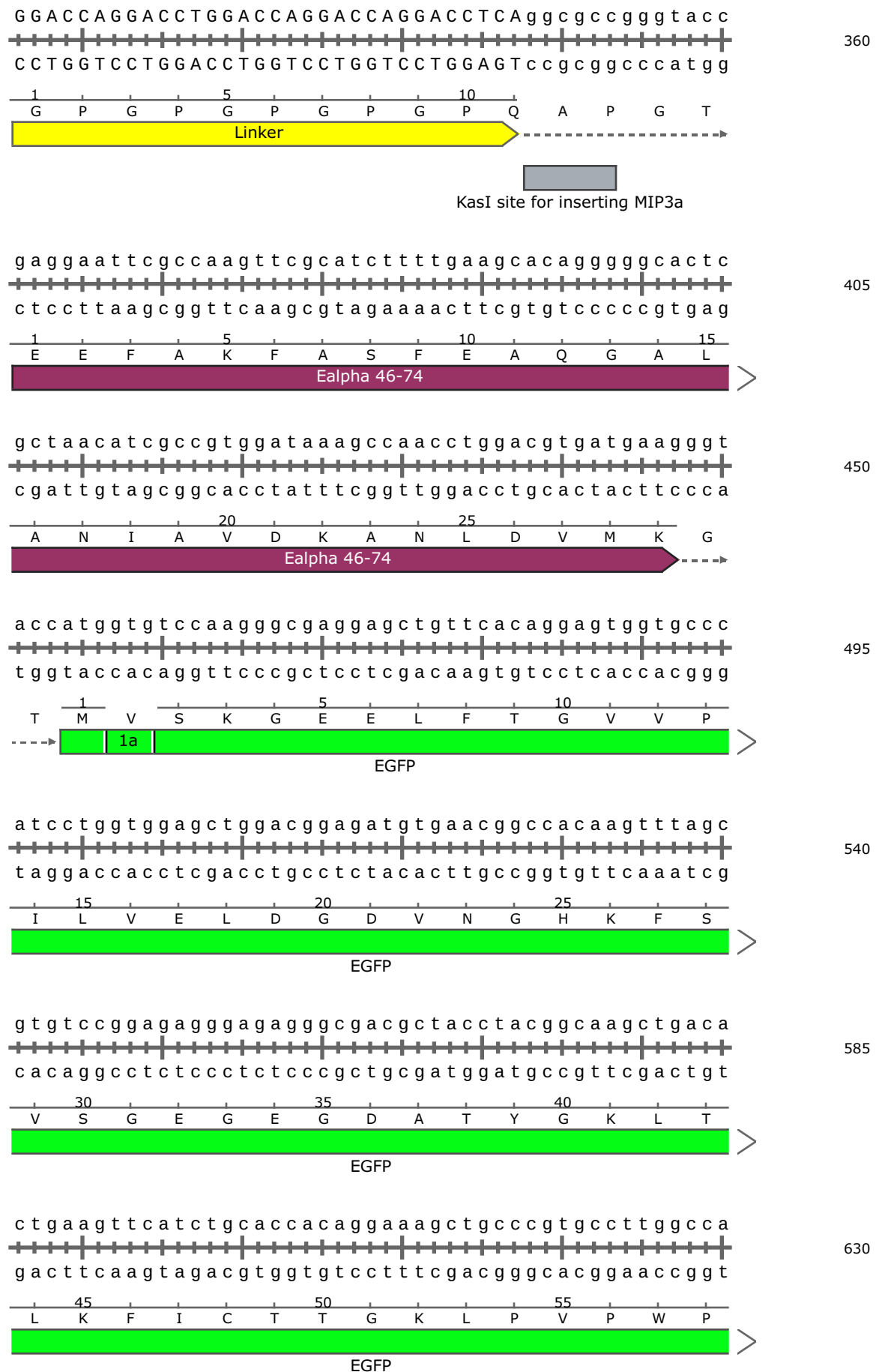

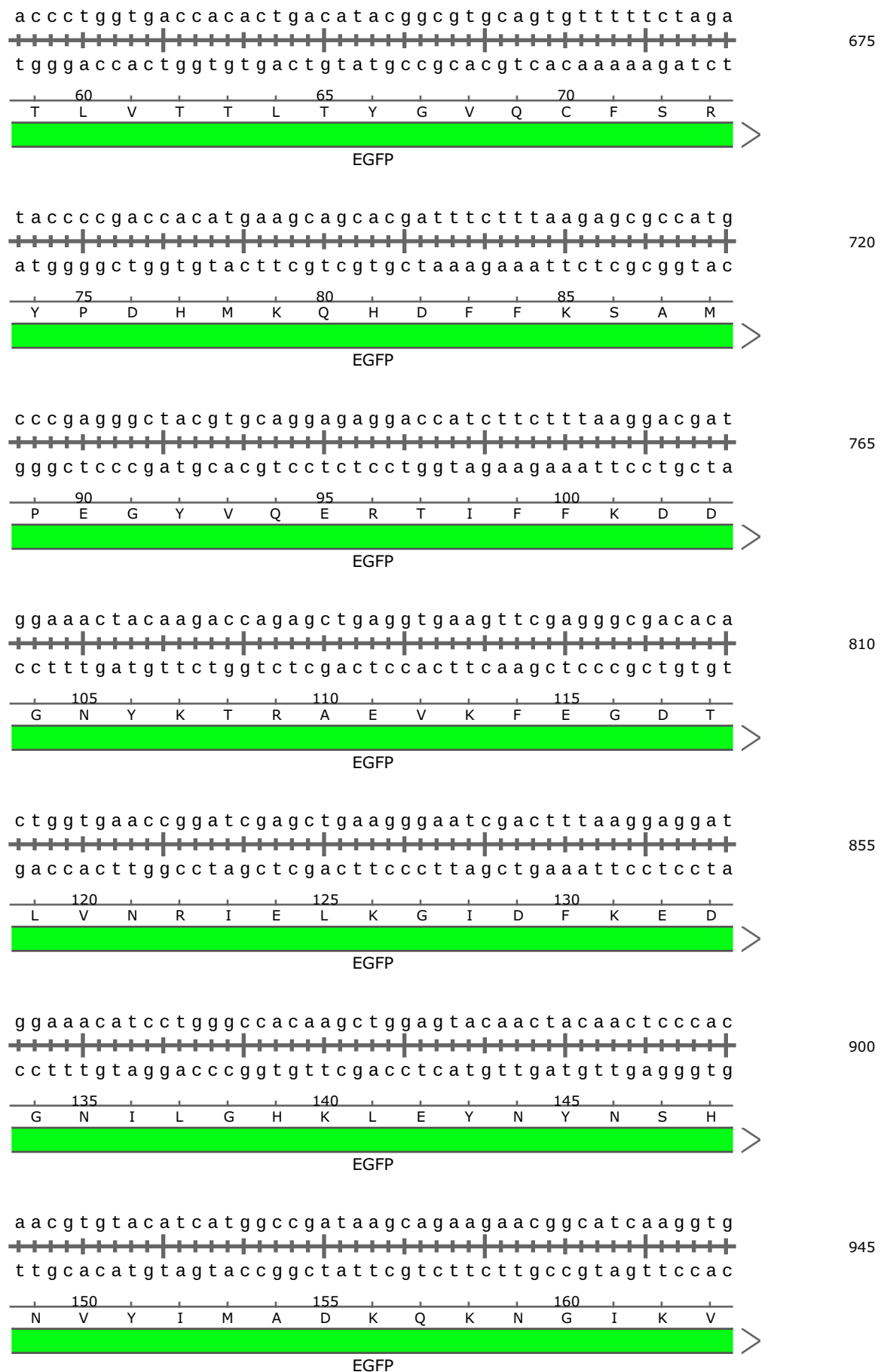

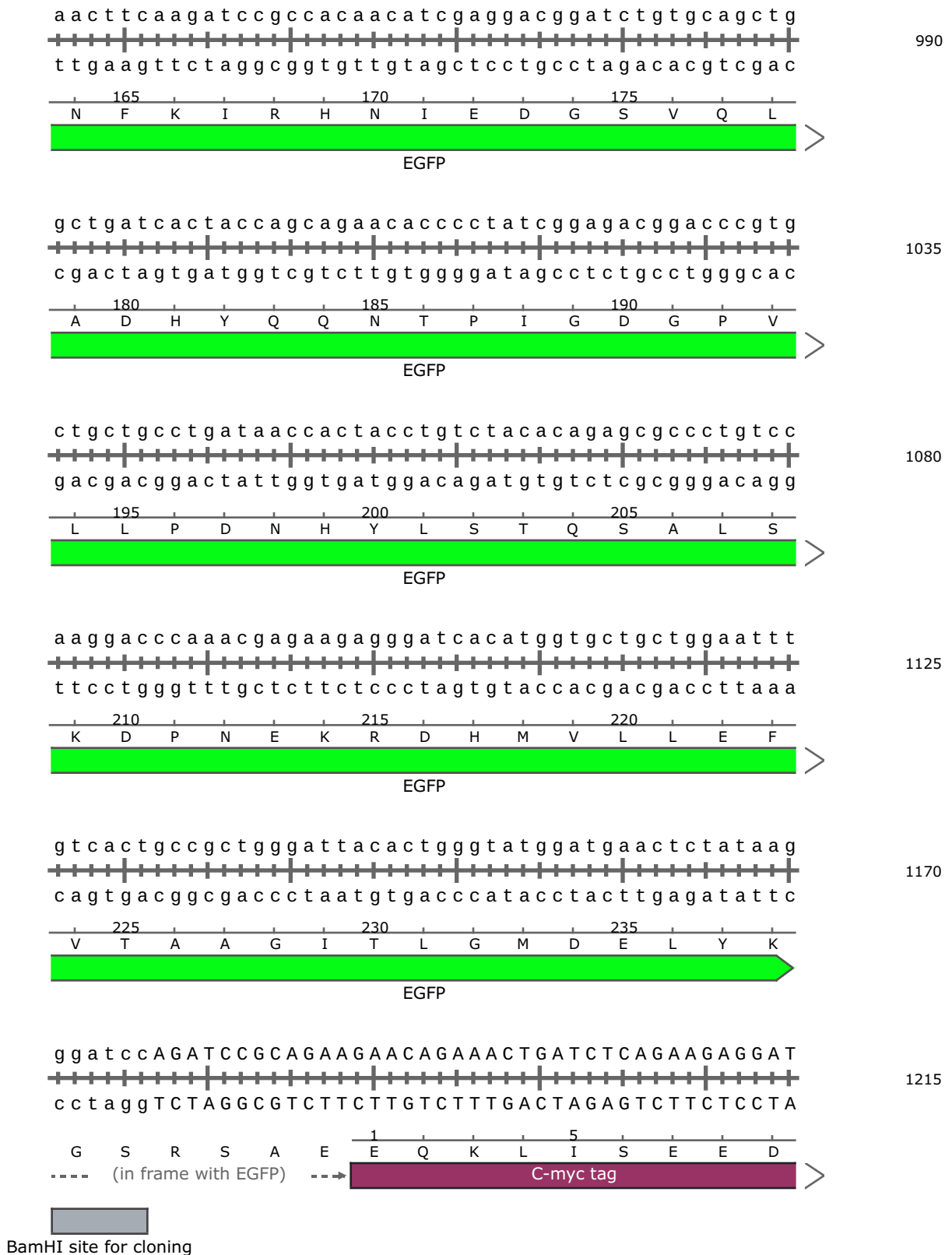

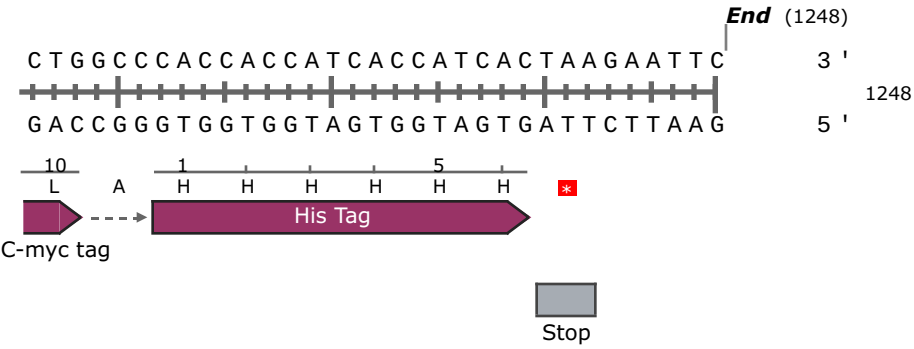

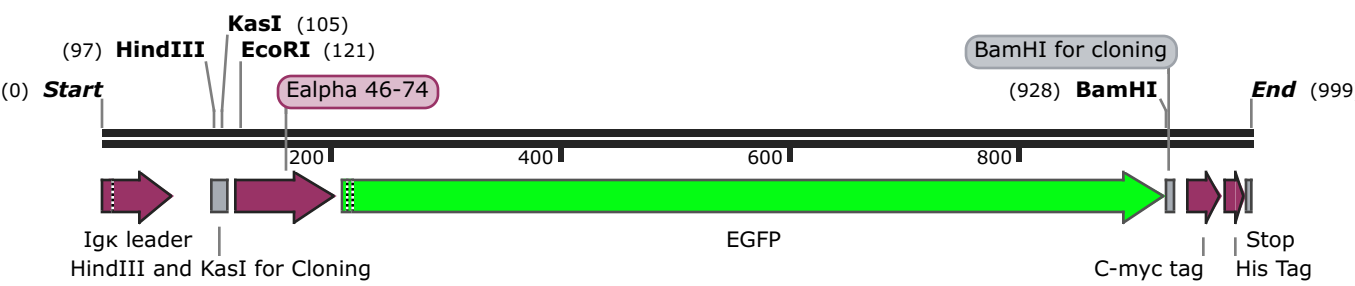

Ea-GFP sequence  
999 bp

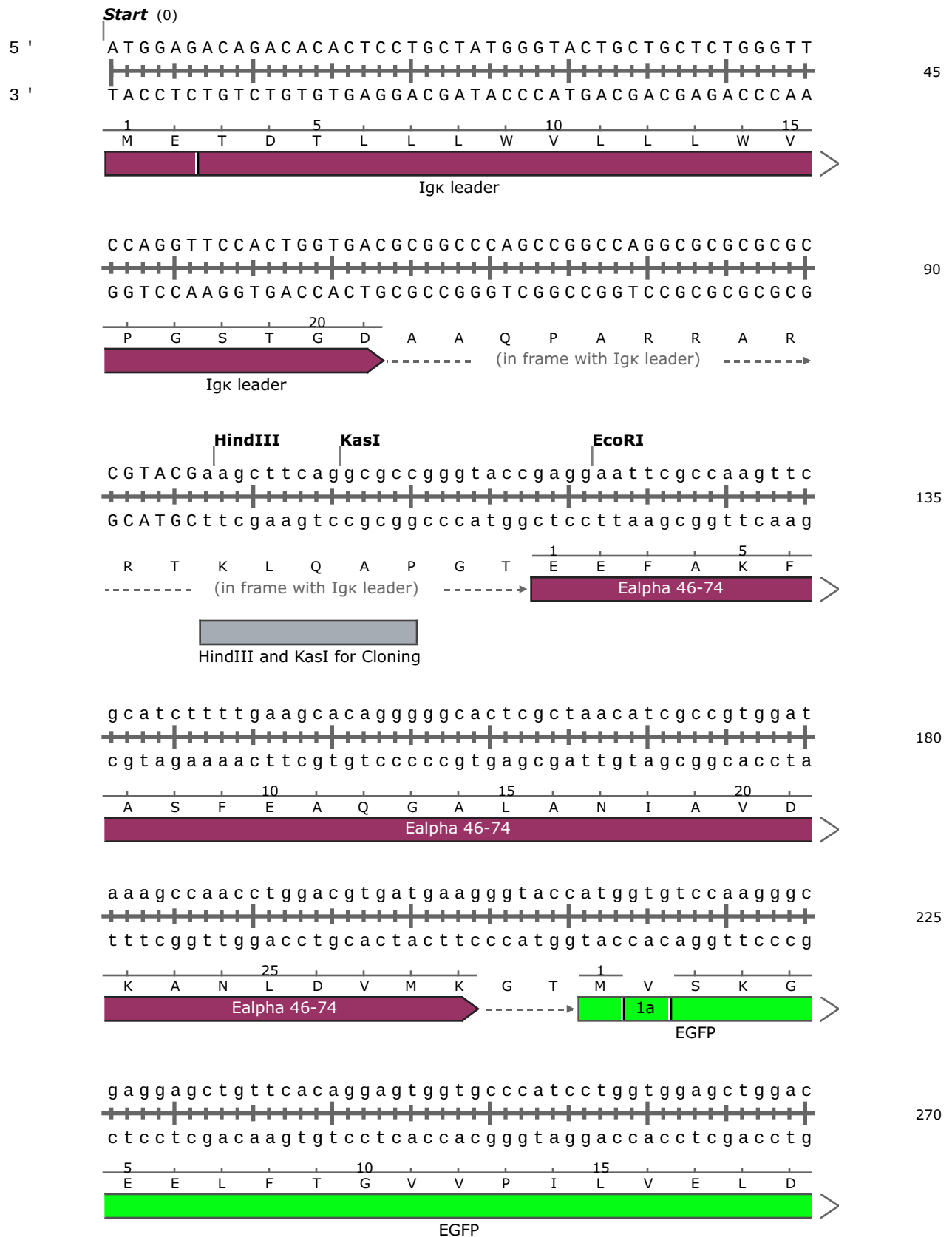

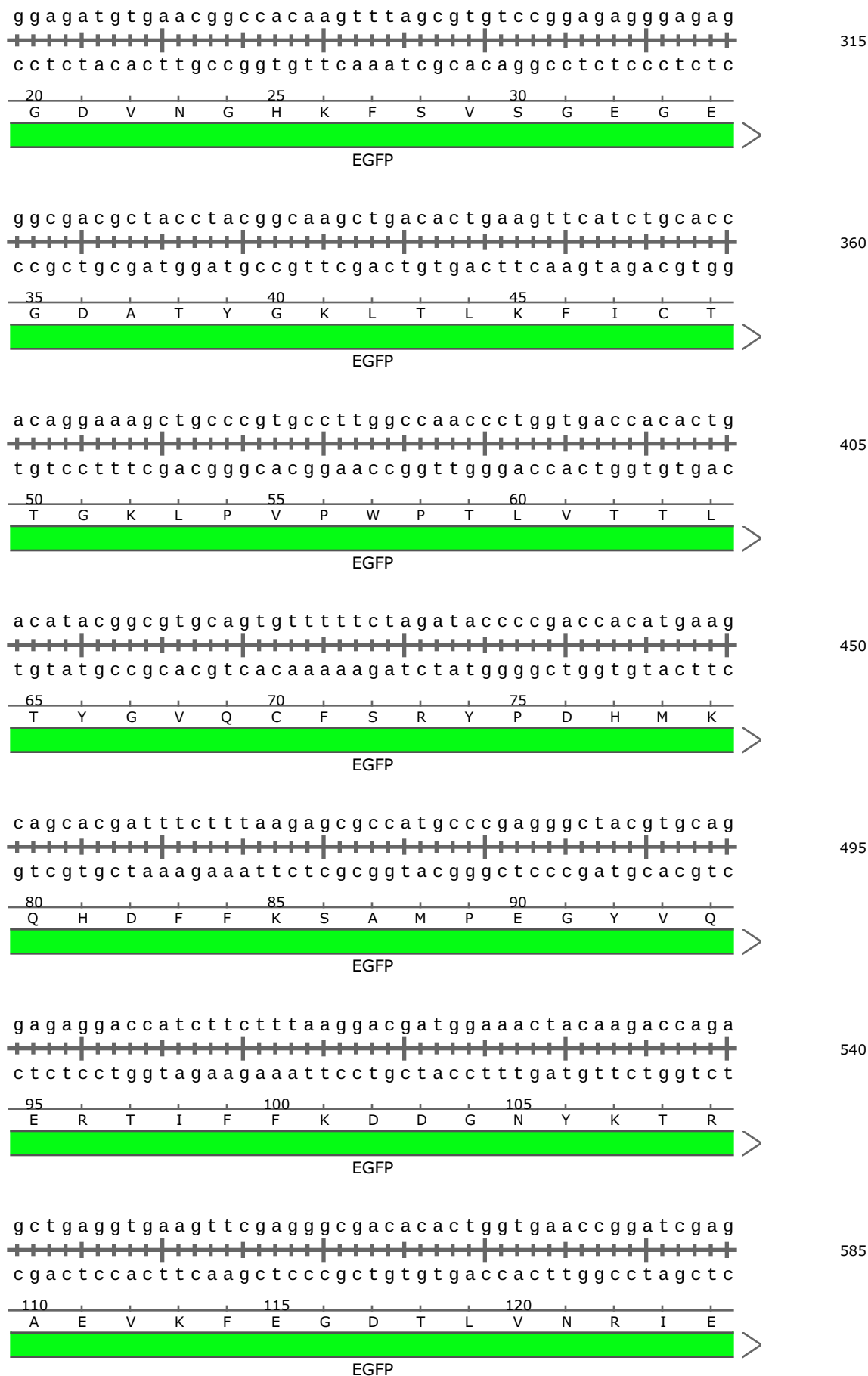

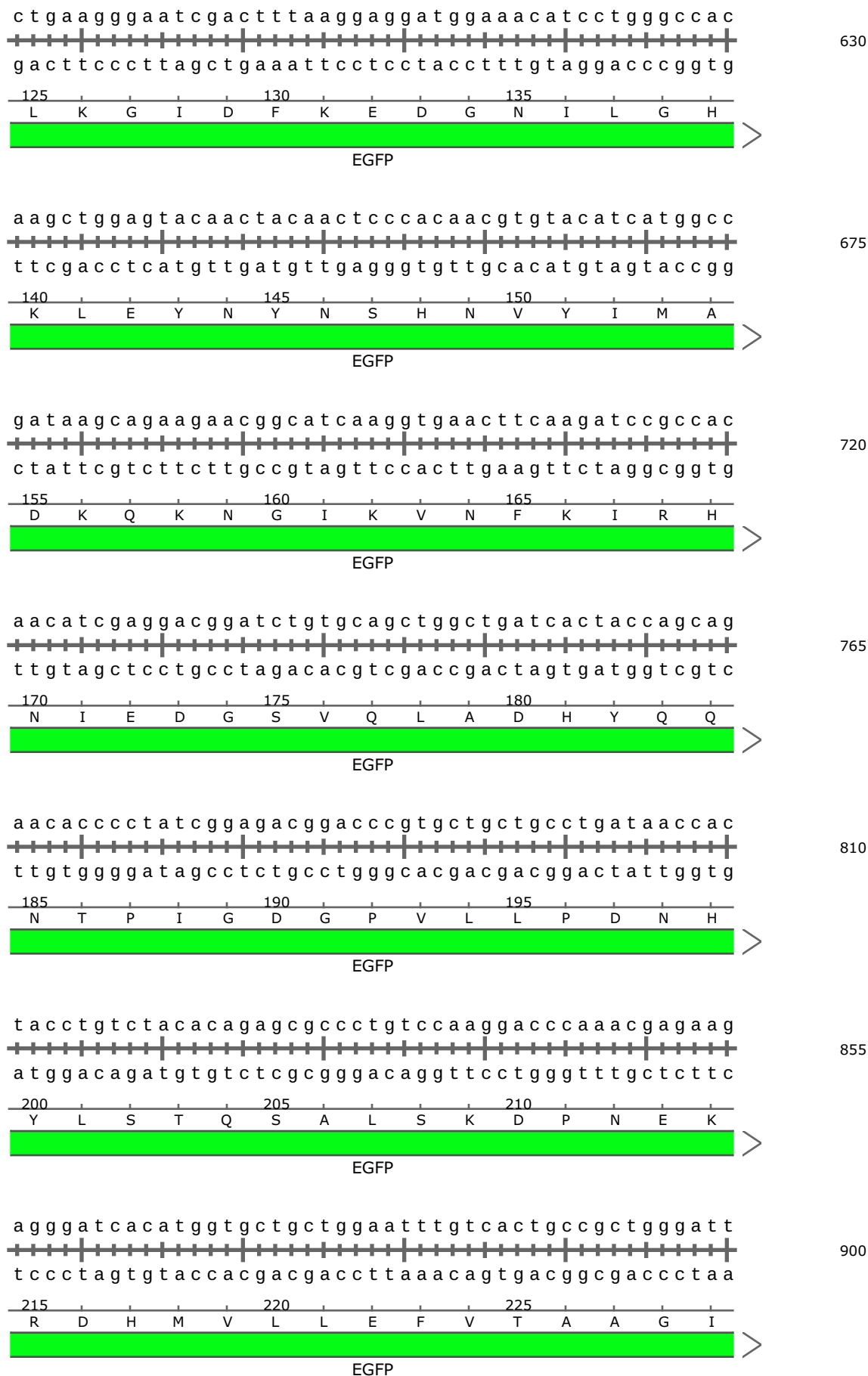

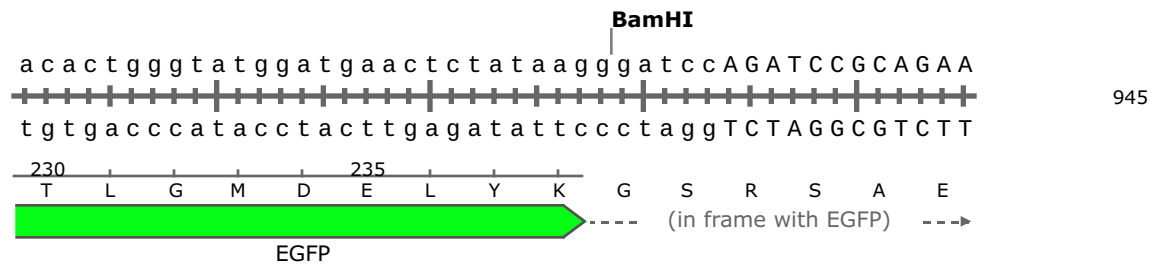

BamHI for cloning

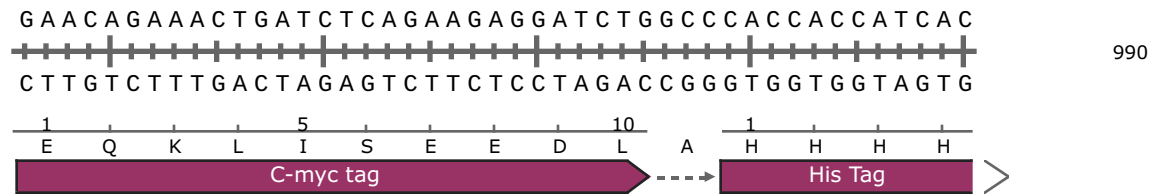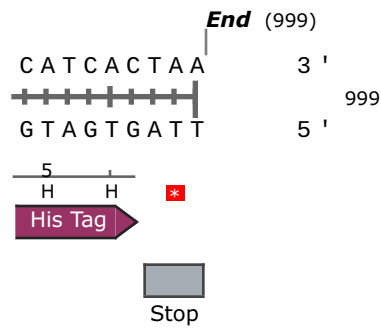

Supplement: Supplementary file 7 — Supplementary Material 1 [file 41598_2025_6532_MOESM7_ESM.pdf]

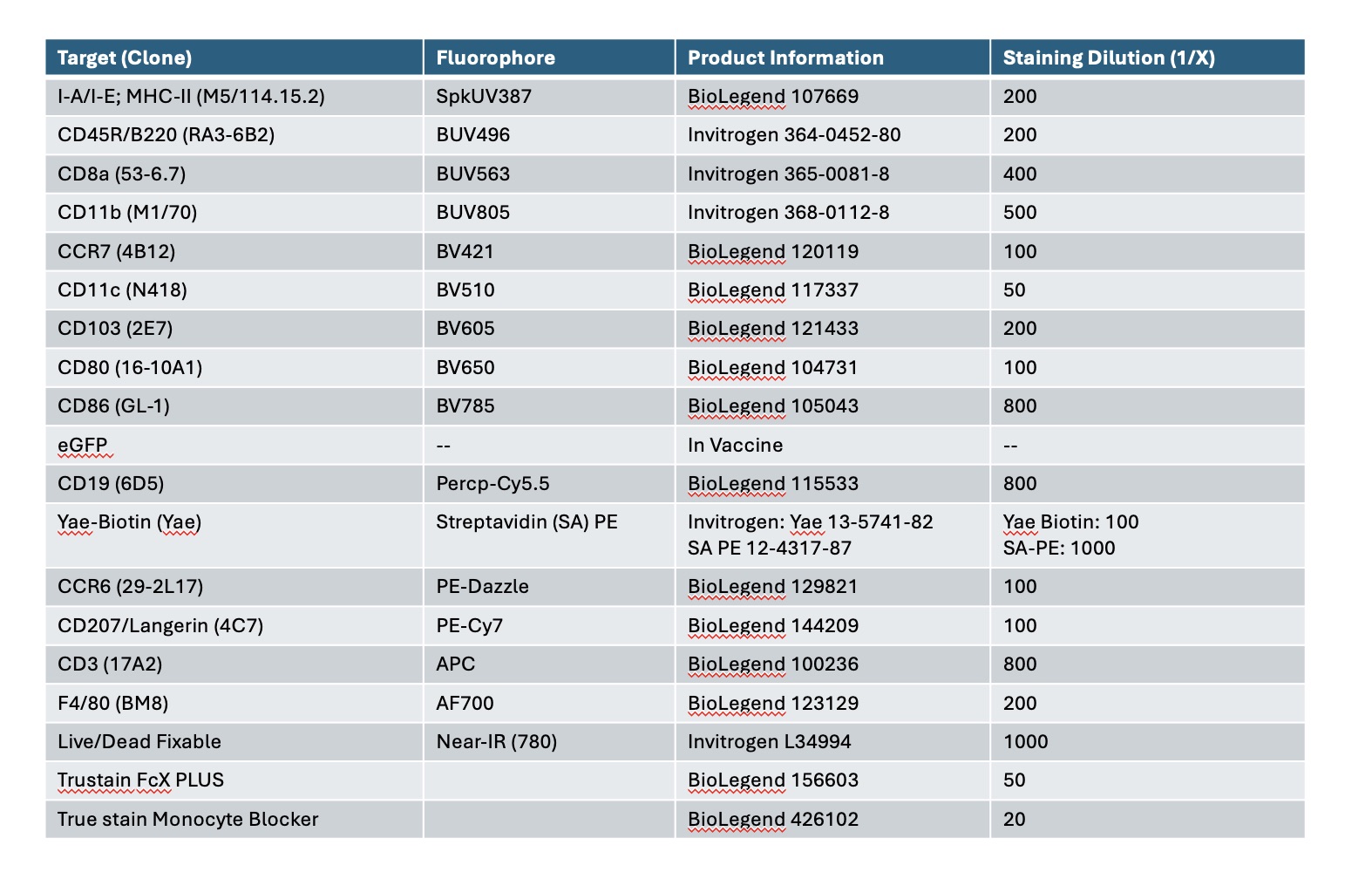

Supplement: Supplementary file 9 — Supplementary Material 9 [file 41598_2025_6532_MOESM9_ESM.jpeg]
