# Supplementary material for: MIP-3α-antigen fusion DNA vaccine enhances sex differences in tuberculosis model and alters dendritic cell activity early post vaccination
Source: Sci Rep. 2025 Jul 1;15:22264. doi: 10.1038/s41598-025-06532-6 (PMC12218986; doi:10.1038/s41598-025-06532-6)
Supplement: Supplementary file 6 — Supplementary Material 4 [file 41598_2025_6532_MOESM6_ESM.docx]

MIP-3α-antigen fusion DNA vaccine enhances sex differences in tuberculosis model and alters dendritic cell activity early post vaccination.

Supplemental Legends:

Supplemental Table 1: Flow Cytometry Antibody Information

Supplemental Figure 1: Vaccine cloning and cell lysate expression analyses. A) EαGFP sequence was cut out of parent pUC plasmid by HindIII and BamHI restriction enzymes and ligated into the pSecTag2b mammalian expression vector. The Figure shows the results of digesting parent EαGFP in pUC and post-ligation EαGFP in pSecTag2b with HindIII and BamHI, showing a band at the expected size of 836bp. B) MIP-3α was cut from another vaccine construct by HindIII and KasI and ligated upstream of the EαGFP. The figure shows a post-ligation confirmation digest with HindIII and KasI with a band at the expected 1175bp. C) HEK293T-cells were transfected with the two constructs and cell lysates were probed by Western blots using anti-c-Myc to detect the construct and anti-β-actin as loading control. C-myc bands were seen at expected sizes of 35kd for EαGFP and 45kd for MIP-3α-EαGFP. Lower band at 30kd likely GFP alone due to alternative transcription, but notably that product was not secreted (Figure 2C). β-actin was seen in approximately equal amounts across the lanes, at expected size of approximately 42 kd.

Supplemental Figure 2: Whole blot and gel images. Dashed boxes are lanes that were cut out of images for reasons displayed. Solid line boxes are the approximate areas of the images presented from: A) the Western blot in Supplementary figure 1; B) the DNA gel image in Figure 2B; and C) the protein gel fluorescent image in Figure 2C. DNA gel images for Supplementary Figure 1A-B were unedited.

Supplemental Figure 3: Screening gates for APCs using a representative sample. All APC flow data first went through the gates of general immune cells, single cells, dead cell exclusion, CD11c+, CD3-, CD19-, and MHCII+

Supplemental Figure 4: APC gates using a representative sample. After gates in Supplemental Figure 2, analyzed APCs went through the following gates. A) Y-Ae gate for all APCs. B) Langerhans cells defined as CD207+ and F4/80+ followed by Y-Ae positivity. The Y-ae gate for these cells was moved further right due to higher background in these cells. C) cDC gates. cDCs were defined as CD207-F4/80- and divided into either CD8+ or CD11b+ populations followed by Y-Ae positivity. CD8+ cDCs were further stratified by CD103.

Supplemental Figure 5: T-cell, B-cell and GFP in the node. After the first three gates in Supplemental Figure 2, overall A) CD3+ T-cells B) CD19+ B-cells and C) GFP from vaccine were assessed. Representative gate shown under the graphs. Data are representative of two independent experiments, n=6-8. nonstatistically significant p-values defined; ns = not significant.

Supplemental Data File 1: Annotated sequences of vaccines used

Supplemental Data File 2: Datasets as presented in the manuscript
